# Regulatory T cell function and engineering for next generation cell therapies

**DOI:** 10.3389/fimmu.2026.1887951

**Published:** 2026-10-19

**Authors:** Nathan W. Zammit, Martina Sassone-Corsi, Payam Zarin, Tingxi Guo, Maegan E. Hoover, Marko Repic, Gene I. Uenishi, Christopher L. Moore, Dalia Gaddis, Mark A. Bach, Chandra Patel, Tiffany F. Chen, Thomas J. Wickham

**Affiliations:** GentiBio Inc., Cambridge, MA, United States

**Keywords:** autoimmunity, cell engineering, clinical trials, Foxp3, IL-2 signaling, inflammation, regenerative medicine, regulatory T (Treg) cell

## Abstract

Regulatory T cells (Tregs) are essential for immune tolerance and tissue homeostasis. Their dysfunction contributes to a broad range of autoimmune, inflammatory, and degenerative diseases, motivating interest in Treg-based therapies. Clinical translation has been constrained by limited stability, persistence, and functional specialization, particularly within inflammatory tissue environments. Emerging evidence reveals that Tregs are not a uniform suppressive lineage but comprise tissue-adapted populations with distinct transcriptional, metabolic, and functional programs. These cells support organ integrity through mechanisms extending beyond immune regulation, including roles in tissue repair, metabolic tuning, and stem cell niche maintenance that significantly broadens their therapeutic potential. Here, we review established Treg biology and key learnings from select clinical experiences using unmodified Tregs. From there, we discuss the mechanisms and challenges limiting efficacy of Tregs, and present how the state-of-the-art engineering aims to enhance Treg durability, stability, and specificity. Finally, we conclude with a forward-looking perspective on how the future of Tregs can expand beyond immune suppression by incorporating recently discovered tissue-specific functions for next-generation therapies targeting a broad range of indications.

## Regulatory T cell biology and clinical experience

The immune system operates under a fundamental dual mandate: it must defend itself against pathogens while maintaining selective non-reactivity to self. Autoimmunity occurs when this delicate distinction fails. At the center of this balancing act are Regulatory T cells (Tregs), indispensable for maintaining immune homeostasis and enforcing self-tolerance (1). Defined by the lineage-specific transcription factor FOXP3 (2–4), Tregs actively constrain autoreactive immune responses and limit inflammation across tissues. Disruption of FOXP3 expression or function leads to systemic immune dysregulation, as exemplified by loss of function mutations that cause polyendocrinopathy enteropathy X-linked (IPEX) syndrome, establishing Tregs as a central and nonredundant pillar of immune tolerance (5, 6).

Tregs arise via two synergistic developmental axes that together generate a regulatory landscape suited to diverse anatomical and immunological contexts. Thymus-derived Tregs (tTregs) emerge early in ontogeny as mature FOXP3^+^CD4^+^ cells following intermediate affinity TCR recognition of self-antigens within the thymus (7–10). This process is governed by Autoimmune Regulator (AIRE)-mediated presentation of tissue-restricted antigens (11). Complementing antigen-driven selection, IL-2–STAT5 signaling acting through the distal *FOXP3* CNS0 enhancer promotes commitment of thymic Treg precursors, and efficient early-life tTreg generation (12). Together, these developmental pathways establish a stable, lifelong foundation of systemic self-tolerance (13–15). tTregs positive for Helios and GATA3 seed the visceral adipose tissue (VAT), skeletal muscle, small intestine, and skin, predominantly during the perinatal period (8, 16–18). In parallel, peripherally induced Tregs (pTregs) arise after birth from naïve CD4^+^ T cells exposed to dietary, commensal, fetal, or environmental antigens in the presence of Treg-promoting signals such as TGF-β and retinoic acid (19–26). This pTreg compartment is particularly important at barrier sites, including the gut, where microbiota-derived cues and metabolites help generate regulatory programs that restrain inflammatory effector responses (22–27). Together, tTregs and pTregs create a multilayered regulatory network that preserves self-tolerance while adapting to local tissue and antigenic contexts (28, 29) (**Figure 1**).

**Figure 1:**
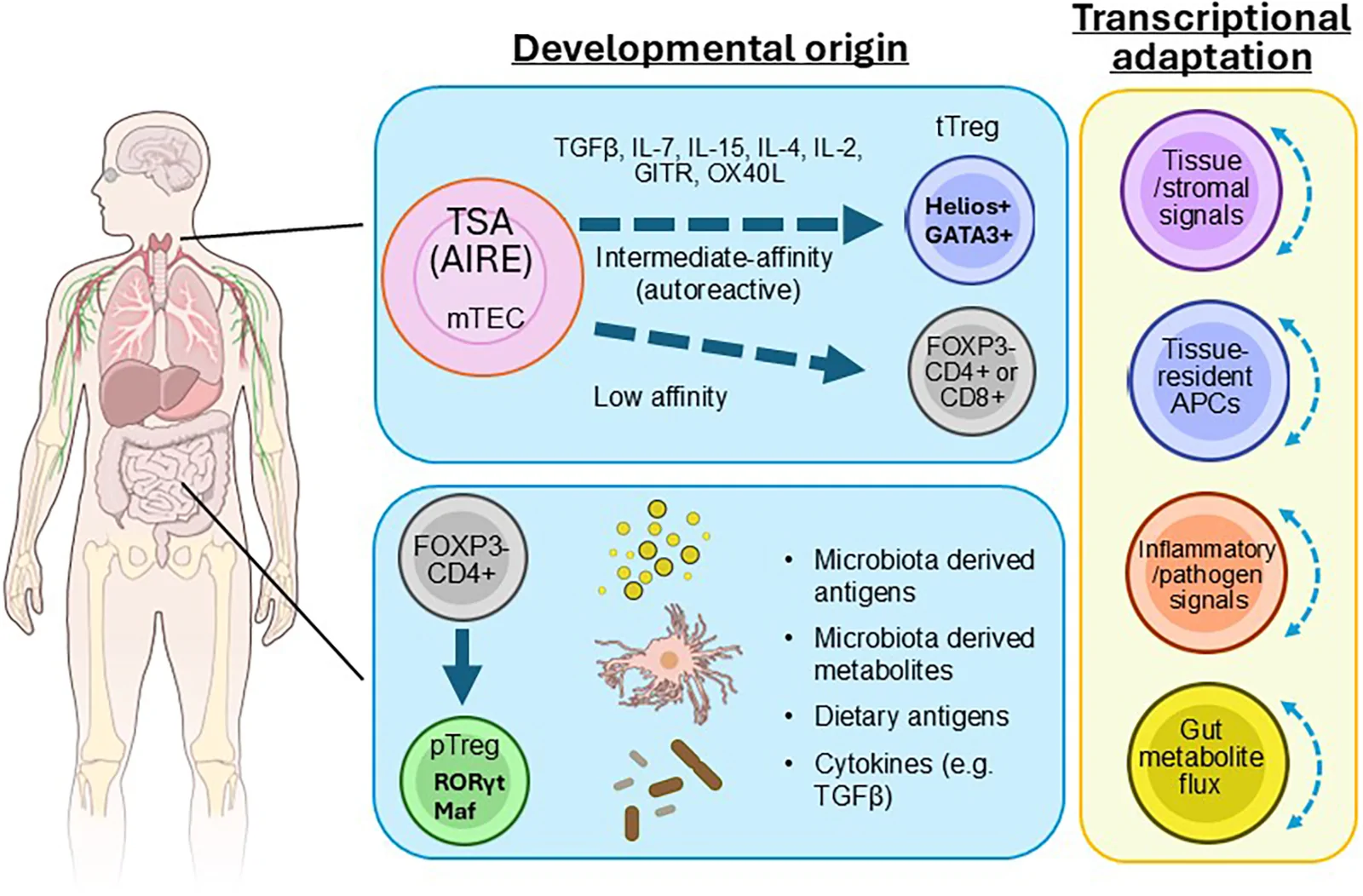
Developmental origin and tissue-driven transcriptional adaptation of regulatory T cells. Treg developmental pathways and tissue-specific cues collectively shape Treg differentiation, survival, function, and distribution across the body. Thymus-derived Tregs (tTregs) originate from the thymus following intermediate-affinity TCR recognition of tissue-specific antigens (TSA) presented by AIRE+ medullary thymic epithelial cells (mTECs), together with signals from TGF-β, γ-chain cytokines and TNFSF family members. These interactions induce stable FOXP3 expression and generate the Helios^+^ GATA3^+^ tTreg lineage (top panel). Low affinity TCR interaction leads to production of FOXP3 negative, CD4+ T cells that can later acquire FOXP3 in the periphery in response to Treg-promoting signals (bottom panel); such as in gut-associated lymphoid tissue, a major site for peripheral Treg induction. Tregs integrate a variety of signals in both lymphoid and non-lymphoid tissues. Inputs from stromal cells, tissue-resident antigen-presenting cells, inflammatory or pathogen-derived cues, and local metabolic conditions collectively fine-tune Treg transcriptional programs. These signals enable Tregs to persist and function across distinct niches, tailoring their roles to site-specific and temporal demands—including regulating immunity during and after pathogen exposure, maintaining tissue tolerance, and supporting local metabolic and homeostatic processes.

Following development, Tregs acquire tissue-appropriate programs through a stepwise process of migration, retention, and local specialization. Lymphoid precursors acquire migratory competence via BATF and IRF4, which cooperate with FOXP3 to prime chromatin accessibility and transcriptional readiness for tissue entry (30). Treg trafficking to non-lymphoid tissues is dictated by tissue-specific chemokine-integrin cues (e.g. CCR4, CCR6 for skin (31), and CCR9 and α4β7 for gut (32)). Tregs then rely on additional mechanisms to maintain tissue retention and egress. CD69-mediated antagonism of S1PR1 provides one layer of control by limiting S1P–driven egress and promoting local retention (19, 20). TCR interaction, local cytokines, metabolites and stromal signals subsequently further define a tissue specific Treg phenotype (**Figure 1**). Representative examples include PPARγ-driven programs in the visceral adipose tissue (VAT), GATA3/RORα in skin (21, 22), and RORγt^+^ programs in the colon. These adaptations are stabilized epigenetically via demethylation of the *FOXP3* conserved non-coding sequence 2 (CNS2)/Treg-specific demethylated region (TSDR), which preserve lineage stability under stress (23–25). Persistence is reinforced by TCR engagement of local self or commensal antigens and tissue-derived survival cues. In particular, IL-33–ST2 signaling sustains Tregs in tissues such as VAT, lung, and muscle (26), whereas pTreg stability in the colon depends on TGF-β and microbiota-derived metabolites, including short-chain fatty acids and bile acids (27).

Within their respective environments, regulatory T cells employ multimodal mechanisms of action, making them a particularly attractive cell therapy approach for intervention in a broad range of autoimmune and inflammatory conditions. Tregs suppress inflammation through a combination of shared core mechanisms and tissue-contextualized suppressive effector functions (reviewed elsewhere (28, 29)). Canonical suppressive strategies include sequestration of limiting cytokines, specifically IL-2 via high-affinity CD25 which both constrains effector T cell expansion and enforces a state of metabolic insufficiency by limiting IL-2–driven mTOR activation and anabolic signaling in responding lymphocytes (28, 33, 34). Additionally, Tregs can hydrolyze ATP via CD39 and CD73 to produce adenosine, a molecule with potent immunosuppressive function, dampening inflammation (29). Critically, Tregs use CTLA-4 to regulate costimulatory ligand availability on antigen-presenting cells; Treg-specific CTLA-4 deletion results in fatal autoimmunity, and a key CTLA-4–dependent mechanism involves capture and internalization of CD80/CD86 via trans-endocytosis, followed by intracellular degradation and reduced CD28-mediated costimulation in a cell-extrinsic manner (35, 36). In selected contexts, Tregs can deploy perforin and granzyme to eliminate pathogenic immune cells, including autoreactive B cells, and release immunoregulatory cytokines such as IL-10 and TGF-β to restore homeostasis (29). In parallel, Tregs integrate environmental signals through receptors such as TNFR2, IL-6R, IL-1RL1/ST2, and IL-18R1, which enhance core Treg programs, reinforce suppressive activity, and restrain local lymphocyte activation while sequestering immune-propagating signals (37–45).

Together, these multimodal mechanisms, supported by decades of preclinical research, provide the rationale for developing Tregs as cellular therapies capable of reinforcing immune tolerance in settings where endogenous regulatory networks are insufficient or where excessive immune activation drives pathology. Early clinical experience with adoptive Treg therapy offers an opportunity to evaluate how clinical success varies across disease settings and to identify where additional engineering or supportive strategies may be needed.

### Adoptive Treg therapy in the clinic: progress and key learnings

Adoptive therapy of autologous polyclonal Tregs has now been implemented in multiple immune-mediated indications, revealing a consistent safety profile but variable efficacy. This section highlights representative studies and key efficacy and persistence findings to illustrate recurring themes in the clinical experience with natural Tregs to date, and how therapeutic activity may vary across disease settings and biological contexts. Across completed trials (reviewed recently (46, 47)), several patterns emerge: clear efficacy signals in graft-versus-host disease (GvHD), inconsistency and modest activity in indications such as solid organ transplantation, and limited durable efficacy in autoimmune diseases.

GvHD represents the clearest success case for polyclonal Tregs. Early-phase studies demonstrated that peri-transplant infusion of autologous Tregs can reduce acute GvHD incidence without increasing pharmacologic immunosuppression (48). These findings have been reinforced in chronic GvHD, where dose-escalation and multi-dose regimens showed improvements in disease severity and enabled tapering of immunosuppressive therapy (NCT02385019; NCT03683498). Importantly, a peri-transplant Treg-containing product, Orca-T (TREGZI) has demonstrated reduced GvHD with preservation of graft-versus-leukemia activity in a randomized Phase 3 study. Specifically, Orca-T increased 1-year survival free from moderate-to-severe chronic GvHD from 38.4% to 78.0%, reduced moderate-to-severe chronic GvHD from 44.0% to 12.6%, and improved GvHD-free/relapse-free survival from 30.9% to 63.1%. This represents one of the strongest efficacy datasets for any Treg modality to date and has recently gained FDA approval (Orca-T platform; NCT03683498) (49). Orca-T is a defined product of CD34^+^ stem cells, Tregs, and conventional T cells (Tconv), where it is proposed that the reduced levels of GvHD observed result from a reduction and delay in the transfusion of Tconv (day 0 infusion of stem cells and Tregs followed by day 2 infusion of Tconv) together with a balanced number of Tregs to control Tconv responses. Additional studies also report signals of clinical remission in steroid-refractory chronic GvHD (SR-cGVHD) (NCT02749084). Collectively, these data indicate that in settings characterized by systemic antigen exposure and accessible immune niches, polyclonal Tregs can exert sufficient suppressive function to restore immune balance. Notably, TCRβ sequencing has demonstrated *in vivo* expansion, lineage fidelity, and persistence of transferred donor-derived Treg clonotypes for up to one year following infusion, both in the context of low-dose IL-2-supported polyclonal Treg therapy and antigen-specific donor Treg products (NCT01795573; NCT06991361; NCT01937468), emphasizing the importance of sustained activation and cytokine support for long-term therapeutic acitvity (50, 51).

Solid organ transplantation, a clinical area of high relevance for Treg biology, has yielded more nuanced and less definitive outcomes. Early pilot studies, mostly focused on kidney transplantation, demonstrated feasibility, safety, and reductions in inflammatory biomarkers (52), while the ONE Study (NCT02129881) which enrolled 130 patients and treated 104 across seven parallel trials, showed no increase in rejection and enabled exploratory tapering of immunosuppression, with acute rejection rates comparable to standard-of-care controls. Indeed, these studies demonstrated evidence of graft-intrinsic immune infiltrates enriched for regulatory markers and transcripts, not present in rejecting grafts or patients receiving Tregs (53). Similar immune infiltrates have also been shown in kidney transplants receiving polyclonal Tregs to treat subclinical inflammation (52). However, subsequent studies have been less encouraging: small Phase I trials reported unclear or equivocal efficacy, and a Phase I/II study found no significant difference versus control despite acceptable safety (NCT02711826) (54). These mixed data suggest that polyclonal Tregs may support immunosuppression minimization, but that optimized dose levels, IL-2 support and persistence remain a challenge. Notably, more targeted approaches, such as ex vivo alloantigen-expanded Tregs in the ONE Study follow-up, have shown durable graft survival with long-term immunosuppression tapering and no rejection over >6 years in select patients (NCT02091232; ONE Study consortium) (55), highlighting the importance of specificity for the control of localized antigen-driven immune responses at the graft interface.

A similar pattern of context-specific activity has been observed in Amyotrophic lateral sclerosis (ALS; aka motor neuron disease or Lou Gehrig’s disease), where infusion of autologous and allogeneic polyclonal Tregs were associated with a slower rate of functional decline across small early-phase studies. Although these beneficial effects were confined to periods immediately post Treg infusion, suggesting limited long-term durability (NCT03241784; NCT04055623; NCT05695521) (56–58). Notably, responses were heterogeneous across participants, with persistent inflammatory signatures associated with continued disease progression despite Treg therapy (57). These outcomes are nevertheless highly promising in such a stringent indication and may highlight key biological barriers including inflammatory-driven instability, and limited CNS/tissue homing. Indeed, Tregs from ALS patients have been shown to be dysfunctional upon isolation, but recover their suppressive properties ex vivo (59).

In contrast, new onset T1D represents an example where monotherapy polyclonal Tregs have not yielded strong clinical benefits in studies reported to date. Early Phase I studies involving fewer than 20 patients suggested signals of preserved β-cell function (NCT01210664), raising initial optimism. However, this was not borne out in larger, controlled settings: a Phase II study (~110 patients; NCT02691247) combining polyclonal Tregs with low-dose IL-2 showed no efficacy in preventing C-peptide decline over one year, despite repeated dosing. Mechanistic insights from parallel trials reveal two likely contributors to this failure. First, IL-2 availability is a limiting factor, as co-administration improved Treg persistence and function (NCT02772679) (60), indicating that survival signals are insufficient *in vivo*. Secondly, it was noted in NCT02691247 that higher fold-expansion during manufacturing correlated with worse clinical outcomes (lower C-peptide levels), suggesting that extensive ex vivo expansion compromises Treg quality. Nonetheless, aside from thus far unsuccessful attempts to address T1D with Tregs alone, a combination treatment with polyclonal Tregs and anti-CD20 antibody impacted residual insulin secretion and duration of remission (61, 62). Together, these findings indicate that only increasing Treg number or dose is insufficient to overcome deficiencies in survival signals, antigen targeting and tissue localization in new onset T1D and potentially other autoimmune diseases.

Taken together, these clinical experiences suggest that to date, while autologous polyclonal Tregs have reported a good safety profile and can be effective in select contexts such as GvHD, their performance has been subpar in many chronic inflammatory or autoimmune settings. Next-generation Treg approaches achieved through optimized functional stability, persistence, and specificity will be discussed in the subsequent section and could catalyze improvements in efficacy.

## Challenges and engineering solutions for Treg therapies

Assessing the emerging clinical experience across a number of indications highlights that while generally safe, the efficacy of Treg therapies remains constrained by context-dependent challenges, including durable persistence and tissue homing (*reviewed elsewhere* (63, 64)). Addressing these limitations will likely require optimization of three interdependent aspects of Treg biology: Phenotypic stability, supportive cytokine signals, and antigen-specific activity.

### IL-2 deprivation, the inflammatory milieu, and gene variants pose barriers to Treg stability & function

Treg dysfunction encompasses impaired suppressive capacity, localization, and signaling defects that compromise immune regulation. Although closely related, Treg dysfunction is conceptually distinct from Treg instability, which is typically linked to a loss of expression of FOXP3-dependent transcriptional and epigenetic programs that maintain lineage identity. Dysfunction may occur in the presence of preserved lineage identity, whereas instability often drives impaired suppressive function and contribution to inflammation or ‘ex-Treg’ states (*reviewed in* (65, 66)). Treg dysfunction and instability can both be amplified by inflammatory cytokines, metabolic stress, and epigenetic dysregulation creating a feed-forward loop that exacerbates pathogenic immune activation and tissue damage in the very environments into which therapeutic Tregs are delivered. Indeed, recent studies demonstrate the critical importance of FOXP3 for Treg stability and function during severe inflammatory conditions, highlighting a vulnerability of therapeutic Tregs in diseased tissue (67). Thus, Treg dysfunction and instability undermine immune tolerance and represent major barriers to Treg-based therapies targeting a wide variety of autoimmune and inflammatory diseases.

A major microenvironmental driver of Treg dysfunction is IL-2 scarcity. Reduced IL-2 availability compromises Treg survival and suppressive function, and can precipitate lineage instability, especially under sustained inflammatory stress (**Figure 2**). IL-2 scarcity may occur through a number of mechanisms including, **1)** IL-2 competition: Although Tregs constitutively express a higher affinity IL-2 receptor complex containing CD25 (68), in immune dysregulated and inflammatory environments activated T cells can upregulate CD25, outcompeting and depriving Tregs of their essential trophic signal (69–71). **2)** Depressed IL-2 production by conventional T effector cells under chronic inflammatory stress: This is best illustrated in the context of systemic erythematosus (SLE) (72), during which sustained TCR/CD3 engagement drives increased cAMP responsive element modulator ɑ (CREMɑ) activity that epigenetically programs IL-17 expression over IL-2 (73–76). This complex re-wiring is facilitated by IL-6 mediated expression of Inducible cAMP early repressor (ICER) converging on SIRT2 deacetylation that limits IL-2 transcriptional accessibility (75, 76). The level of CREB mediated IL-2 suppression by effector T cells is also tuned by the dysregulated expression and activity of key kinases and phosphatases such as PP2A and its subunit regulators or calcium/calmodulin-dependent protein kinase IV (CaMK4) (77–79). Parallel overactivation of T cell mTORC1 pathway due to inflammatory mediators such as leptin can also promote a Th17 phenotype at the expense of Treg stability (80–82). **3)** Treg sensitivity and response to IL-2: In addition to extrinsic pressures mediated by effector T cells, Treg-intrinsic interruption of IL-2-CD25 signaling can accelerate dysfunction by either disabling normal sensitivity and response to IL-2 at the level of the CD25 receptor, or via transcriptional regulators (83–85). Dysregulated kinases and phosphatases (e.g. PP2A), which under normal conditions support Treg function (77, 86), can amplify Treg dysfunction through a variety of mechanisms such as insufficient balance of extracellular metalloproteinases such as ADAM9 essential for liberating TGF-β (87) or ADAM10 that impairs IL-2 signaling via cleavage of the IL2R-β receptor (88).

**Figure 2:**
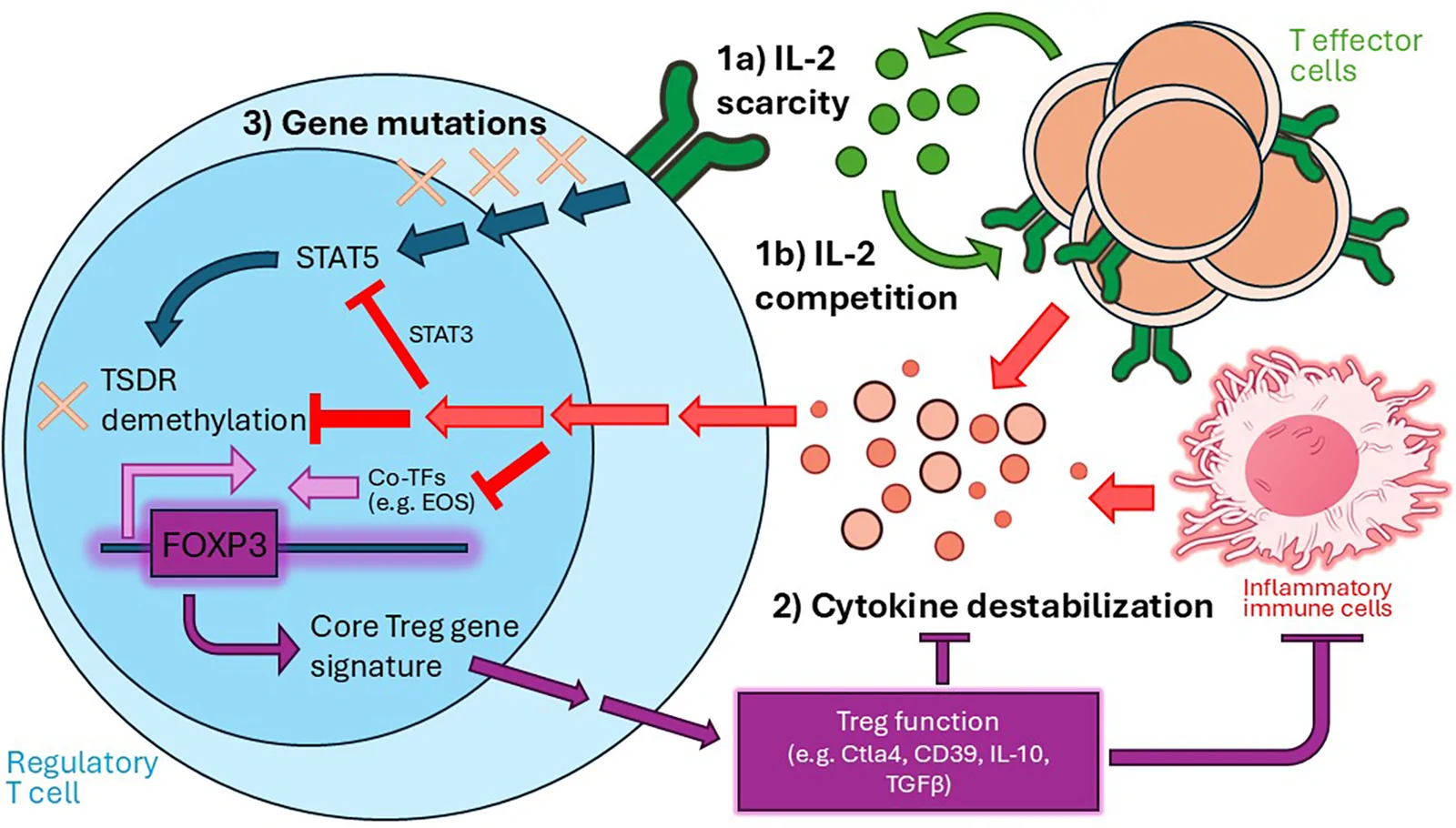
Treg instability and dysfunction. Treg differentiation and function is supported by IL-2 signaling (blue arrows). This essential pathway may be impaired as Tregs traverse lymphoid and tissue environments, challenging their stability. First, IL-2 availability (green) is often scarce (1a) in tissue environments limiting Treg persistence. Additionally, during immune activation, competition for IL-2 by activated T cells (1b) limits IL-2 availability to Tregs. Reduced IL-2 signaling increases susceptibility of Tregs to cytokine destabilization (red; 2), mediated by cytokine signals (e.g. IL-6, TNF, IL-1β) relayed by their respective Treg expressed receptors that can activate degradation or signaling pathways that block IL-2 signaling. Lastly, gene mutations (3; orange) in genes that make up IL-2 signaling pathway can also increase susceptibility of Tregs to instability and dysfunction in IL-2 scarce and inflammatory environments.

As IL-2 signaling diminishes, STAT5 activation declines, leading to compromised maintenance of FOXP3 expression, including re-methylation of the Treg-specific demethylated region (TSDR). These events can erode the expression of a ‘core set’ of genes integral to Treg function, including *IL2RA* (*CD25*) and *CTLA4* (71, 89), driving a loss of regulatory capacity and leaving Tregs vulnerable to inflammatory reprogramming by cytokines such as IL-6, IL-1ß and TGF-ß (90–94). For example, *FOXP3*^+^ Tregs within inflamed joints have been shown to convert to Th17-like “ex-Tregs” expressing IL-17 or RANKL, upon prevention of IL-2-CD25 signaling that precedes *FOXP3* downregulation (65, 95–97).

Intrinsic defects in Treg functional pathways can also predispose to instability (**Figure 2**). Similar to variants in genes encoding components of IL-2 signaling and sensing, variants that suppress Treg function reduce their durability in inflammatory environments. Unsurprisingly, mutations in genes encoding Treg suppressive proteins such as LRBA and DEF6, which regulate CTLA-4 trafficking, are linked to autoimmune cytopenias and SLE (98, 99). Additionally, polymorphisms in PTPN2 and TNFAIP3, which modulate TCR and NF-κB signaling, can destabilize Tregs and predispose individuals to SLE, T1D, Behcet’s and Hashimoto’s thyroiditis (100–103). Such environmental and genetic mechanisms of instability and dysfunction contribute to self-reinforcing inflammatory reactions that have been implicated in a number of inflammatory and autoimmune diseases, such as rheumatic diseases (96, 104), liver disease (105, 106), inflammatory bowel disease (107, 108), multiple sclerosis (MS) (109–112), SLE (72, 113) and T1D (114–118) (*also recently reviewed* (119).

T1D in particular, shows a strong association with mutations in IL-2RA (114, 120–122), and clinical studies have shown reduced suppressive ability of Treg cells in patients with T1D (123, 124). These defects converge on impaired IL-2/CD25 signaling consistent with the mechanisms described above (115, 116, 125). Supporting this, blockade of CD25 via anti-CD25 therapy in solid organ transplantation associated with increased incidence of diabetes in treated recipients (126, 127). Mouse studies using non-obese diabetic (NOD) mice further show that augmentation of IL-2 signaling can prevent and reverse disease, by preventing Treg cell death upon activation, maintaining an appropriate Treg:T effector balance, and restricting Treg plasticity/instability (115). Tang et al. (2008) (117) showed these effects to be prominent in Tregs within the inflammatory environment of the pancreas, rather than distal lymph nodes, and are driven by defective IL-2 signaling. Together these data provide strong evidence that Treg dysfunction or instability contribute directly to T1D pathogenesis, positioning Treg-based replacement therapy as a compelling therapeutic strategy.

The above summary highlights the pathogenic Treg instability mechanisms across a number of diverse tissue systems. However, for many conditions, the role of Tregs in disease penetrance is still unknown, or just emerging (101, 128). In one example, patients with ALS show reduced CD4^+^CD25^+^FOXP3^+^ Tregs and lower FOXP3 gene expression in peripheral blood, with counts inversely correlated with disease severity, suggesting early Treg instability may impair neuroprotection (59, 129). A pilot ALS clinical trial infusing autologous Tregs in combination with IL-2 demonstrated improved outcomes compared to previous trials that infused only Tregs, underscoring the central importance of IL-2 for Treg stability and function (57). These data, and others demonstrating a Treg imbalance in patients with neurological indications, reflect an emerging role for Tregs in neuroinflammatory disease (130, 131).

In sum it is clear that environmental and polygenic factors, often occurring simultaneously, overwhelm the ability of endogenous Tregs to control aberrant immune responses within tissue specific niches (**Figure 2**). This is a particular challenge when attempting to utilize these same cells to treat inflammatory disease conditions. Notably, Treg dysfunction does not necessarily represent an irreversible state. In a reversible Foxp3-deficiency model, restoration of Foxp3 expression in mice with severe systemic inflammation re-established Treg suppressive activity, normalized immune activation, and reversed otherwise fatal autoimmunity, demonstrating the profound capacity of functional Tregs to restore immune homeostasis even after disease is established (132). Understanding the drivers of Treg destabilization and dysfunction will assist in the design of tailored and durable therapeutic strategies. Such approaches could include targeting endogenous Tregs that are extrinsically impaired through biologic or *in vivo* gene-editing technologies, or alternatively, the administration of *ex vivo* expanded or engineered autologous or allogeneic Treg populations.

### Engineering Treg stability

Given the importance of stable FOXP3 expression, multiple approaches have been described to enhance the stability of this transcription factor. Forced expression of FOXP3 has shown success in converting readily available Tconv to stable Tregs or used to reinforce FOXP3 stability in natural Tregs. Viral transduction of ectopic FOXP3 is a common approach in the field (48, 133–136). An ongoing clinical trial (NCT05241444) is investigating autologous LV-FOXP3 transduced CD4 T cells, in patients with IPEX syndrome, with preliminary data indicating maintenance of a Treg phenotype in engineered cells (137). In contrast to semi-random integrating viral vectors, a site-specific approach where a strong constitutive promoter is knocked-in at the endogenous *FOXP3* locus also facilitates stable Tconv-Treg conversion (138–140). With either approach, the converted T cells gain the phenotypic and functional attributes of Tregs marked by elevated expression of suppressive molecules such as CD25, CTLA4, LAP/GARP, and reduced production of inflammatory cytokines such as IL-2 and IFN-γ. Of note, the promoter knock-in approach achieves higher FOXP3 expression than lentiviral transduction methods (138), which could be important given the dose-dependent relationship between FOXP3 protein levels and Treg function (141). Conversely, select patients with loss-of-function mutations in *FOXP3* exonic sequences (142) will require a gene replacement/correction approach. Emerging clinical data for FOXP3-engineered and Tconv-converted Tregs will be informative on the long-term functional stability of this approach compared to natural Tregs with native FOXP3.

Beyond direct modulation of FOXP3, early discovery work identified that other genetic and epigenetic targets can be overexpressed or ablated to stabilize FOXP3-dependent Tregs, or aid in the Tconv-Treg conversion. Using a retroviral system, Tconv engineered to co-express FOXP3 and Helios, a Treg-associated transcription factor enriched in tTregs (143), slowed xenogeneic GvHD more effectively than cells expressing FOXP3 alone (144). The mechanism underlying this improved function is unknown. Notably, no major difference in expression of Treg markers measured by flow cytometry (CD25, CD73, CCR4, IL-10) or global gene expression were detected between FOXP3 and FOXP3+Helios overexpression. Although the precise role of Helios remains debated (143), these findings highlight the important role FOXP3 cofactors play during Tconv-to-Treg reprogramming. Supporting this concept, very high expression of FOXP3 by knock-in of an MND promoter upstream of the endogenous promoter is sufficient to drive the upregulation of several FOXP3 cofactors, including Eos (140) that may explain the success of this approach. Eos has been shown to be critical for the function and stability of Tregs (145) and can cooperate with Helios and FOXP3 to bind directly to regulatory regions of the CD25 (IL2RA) locus (146). Consistent with this network view, FOXP3-dependent chromatin remodeling appears to occur largely at sites not directly bound by FOXP3, in part through tuning trans-acting intermediaries such as TCF1, arguing that durable Treg engineering may require reinforcement of FOXP3-coupled transcriptional circuitry rather than FOXP3 expression alone (27, 147). These results underscore the impact FOXP3 reprogramming approaches have on the induction of a cooperative transcriptional network, which are essential to enhance Treg conversion and stabilize function (148).

Additional molecular pathways also influence Treg stability. Overexpression of ARKADIA, an E3 ubiquitin ligase that targets Treg destabilization factor SKI for degradation, enhances Treg phenotypic and functional stability under stress conditions (149). A targeted CRISPR screen of nuclear regulators of Foxp3 expression in mice identified Usp22, a de-ubiquitinase, as a positive regulator of Foxp3 through direct post-translational regulation. Knockout of USP22 in human Tregs resulted in reduced FOXP3 expression, confirming cross-species biology (150). A separate genome-wide loss-of-function CRISPR screen in human inducible Tregs (iTregs), identified the RBPJ-NCOR complex downstream of Notch signaling as a context-dependent modulator of FOXP3. RPBJ-deficient iTregs exhibited superior suppressive function in a xenogeneic GvHD model compared to control iTregs and reduced methylation at the CNS2 region of the *FOXP3* gene, a hallmark of natural Tregs (25, 151). These studies indicate that other molecular regulators may be manipulated to further enhance functional stability. Identifying the minimal set of molecular payloads for cell engineering, while achieving the necessary Treg stability, will be crucial to the practical implementation of methods involving gene modifications.

In addition to molecular engineering approaches, recent advances in reprogramming conventional CD4 T cells into stable iTregs using small molecules and biological factors have been reported (152, 153). Such methods could avoid the need for genetic modifications. Historical iTreg approaches relying solely on anti-CD3/CD28 and TGF-β stimulation suffered from poor stability, with the converted Tregs possessing methylated CNS2 (154–156). Three epigenetic-modifying chemicals (sodium butyrate, UNC0646, and vitamin C) in combination with TGF-β were reported to generate iTregs with greater stability, demonstrated by CNS2 demethylation and maintenance of FOXP3 under inflammatory culture conditions, resulting in higher suppressive capacity than conventional iTregs (152). Another approach involves stimulating human Tconv with anti-CD3, but not anti-CD28, in the presence of TGF-β, CDK8/19 inhibitor senexin A, anti-FasL, and ascorbate, which generated iTregs with more consistent CNS2 demethylation, as well as higher frequency of FOXP3, CD25, and CTLA4 expression (153). This approach builds on the previous work showing that CDK8/19 inhibition facilitated Foxp3 expression in murine T cells (157). The use of vitamin C in both methods is aligned with the reproducible observations of vitamin C promoting Foxp3 expression in multiple studies (158–160), albeit largely limited to murine systems. Collectively, emerging molecular and biochemical strategies described above can potentially generate Treg products with the desired phenotype and stability, as an enhancement of, or alternative to, natural Tregs for therapeutic applications. The relative stability of induced and genetically reinforced Tregs will require direct comparisons in future studies.

### Engineering supportive cytokine signals to support clinical Treg persistence

Tregs critically depend on cytokine signals for survival and function, with IL-2 as the prototypic cytokine signal (71, 161, 162). Low-dose IL-2 can preferentially support Treg over Tconv cells given that Tregs express greater levels of the high affinity receptor CD25. Balancing the narrow therapeutic index with off-target immune activation of natural IL-2, however, is clinically challenging (60, 163, 164). To broaden its therapeutic window, recombinant IL-2 has been modified with PEGylation or site-specific mutations to further bias binding to CD25. These strategies have shown promising initial clinical results and serve as an important proof-of-concept for Tregs as a therapeutic strategy (165–169). One indication in which PEGylated IL-2 biologics have shown recent clinical success is atopic dermatitis (AD), where rezpegaldesleukin (REZPEG) significantly improved Eczema Area and Severity Index (EASI) for moderate to severe AD in a Ph1 and 2 study (169). In addition to their clinical potential as a monotherapy (165, 170), the favorable safety profiles and demonstrated capacity to expand functional Tregs in humans support the utility of these modified IL-2 biologics as part of the toolkit for next generation Treg therapies. However, recombinant IL-2 approaches are unlikely to benefit patients with genetic defects in endogenous signaling components (84).

Engineered orthogonal cytokine and receptor pairings have been explored to selectively support therapeutic cells without activating endogenous immune cells. These transgenic systems also overcome intrinsic dysfunctions at the receptor level required for survival signals. Sockolosky et al. described a mutated IL-2 that does not activate wild-type receptor but engages a compatibly mutated IL-2Rβ, albeit with lower functional activity than the wild-type IL-2/receptor system when stimulating T cell responses *in vitro* (171). Building on this platform, the Tang group introduced the orthogonal IL2/Rβ in murine Tregs in a tethered manner, where the orthogonal IL-2 is covalently linked to the orthogonal IL-2Rβ (172). In a BDC2.5 T cell transfer model of diabetes, Tregs with tethered orthogonal IL-2 prevented disease onset significantly better than control Tregs. Notably, the group found that using the soluble variant of the orthogonal IL-2 to support orthogonal IL-2Rβ-engineered Tregs did not provide significant improvement (172), which would have provided inducible control over the IL-2 signaling compared to constitutive signaling of the tethered version. This highlights one challenge of modifying natural cytokine systems to achieve orthogonality, controllability, and robust biological activity. Additional challenges may be related to the emergence of complex tissue specific cytokine networks where fine-tuned integration of signals is a driver of specialized Treg function.

To avoid crosstalk with the native IL-2/receptor, one strategy is to design a synthetic cytokine-receptor system to deliver orthogonal IL-2 signals to Tregs. In one example, a chemically inducible signaling complex (CISC) was generated by fusing the rapamycin-dimerizing domains FKBP12 and FRB (174) to the intracellular signaling domains of IL-2 receptor subunits CD132/IL2Rg and CD122/IL2Rβ (173). Upon rapamycin treatment, CISC-transduced human T cells expand *in vitro*, leading to selective enrichment of engineered cells. When CISC is knocked-in upstream of the endogenous *FOXP3* gene, engineered human Tregs are produced with high purity and persist at higher levels *in vivo* over time with rapamycin support versus vehicle treatment (173). By leveraging the heterodimeric nature of CISC, the molecule can be split across two vectors to facilitate enrichment of dual-engineered cell products; one subunit can be encoded upstream of *FOXP3* and another encoded along with an antigen-specific receptor. This approach has enabled the generation of antigen-specific engineered Tregs that are readily manufacturable and can benefit from CISC support in a controllable manner for persistence in clinical settings (139, 140, 175). This strategy is currently being evaluated in a clinical trial for T1D (NCT06919354), which will shed light on the effectiveness of such orthogonal and inducible cytokine support systems on the durability of Tregs in patients.

### Antigen specificity and activation

Autologous polyclonal Tregs have shown limited clinical efficacy in tissue-specific autoimmune diseases (176). Engineering antigen specificity could provide an opportunity to improve Treg efficacy by 1) modulating function through TCR-mediated activation (177), and 2) localizing activation to where pathological antigens are being presented to minimize systemic immune suppression. Analogous to ex vivo engineered Tconv therapies for oncology and infectious diseases, Tregs can express a variety of recombinant antigen receptors (**Table 1**). The discussion here will be focused on antigen specificity engineering demonstrated with human Tregs and antigen systems, mostly at the preclinical stage unless noted otherwise. Expression of recombinant TCRs targeting autoantigens presented by HLA class II allows therapeutic Tregs to suppress T effector responses initiated by professional antigen-presenting cells (APCs) (140, 178–180). HLA-restricted T1D-associated epitopes have been extensively characterized (197). Using clonal TCRs specific for IGRP-derived epitopes presented by DRB1*04:01, antigen-specific engineered Tregs were generated using the *FOXP3* knock-in approach described above (139, 140, 179). Antigen-specific engineered Tregs not only suppressed T cell responses targeting the same epitope, but also demonstrated bystander suppression of polyclonal effector T cell response against multiple disease-associated epitopes (140). Bystander suppression was also demonstrated using natural Tregs transduced with a DR4-restricted GAD65-specific TCR (178). Suppression of bystander pathological T cells is a necessary feature for effective control of autoimmune diseases such as T1D, where multiple epitopes are typically targeted by autoreactive responses (198). In addition to T1D antigens, human Tregs have been engineered with recombinant TCRs in preclinical settings to target HLA class II-restricted epitopes derived from myelin basic protein (136, 180) and PDC-E2 (175) with the potential to treat multiple sclerosis and primary biliary cholangitis, respectively.

**Table 1:** Approaches to rewire antigen specificity for Treg activation.

Targeted antigen	Recombinant receptor	Applicable indication	References	Registered clinical trials (as of publication)
DR4/GAD65	TCR	Type 1 diabetes	Yeh et al., 2017 (178)	
DR4/IGRP	TCR	Type 1 diabetes	Yang et al., 2022 (179), Uenishi et al., 2024 (140)	NCT06919354
DR15/MBP	TCR	Multiple sclerosis	Kim et al., 2018 (180)	
DR4/MBP	TCR	Multiple sclerosis	McGovern et al., 2022 (136)	
DRB4/PDC-E2	TCR	Primary biliary cholangitis	Tewari et al., 2024 (175)	
DR1/FVIII	TCR	Immunogenic responses to FVIII replacement therapy	Kim et al., 2015 (181)	
HLA-A2	CAR	HLA-A2 mismatched transplant	MacDonald et al., 2016 (182), Henschel et al., 2023 (134), Tripathi et al., 2025 (183)	NCT05234190, NCT04817774
Citrullinated-protein	CAR	Rheumatoid arthritis	Raffin et al., 2019 (184)	NCT06361836, NCT06201416
MBP or MOG	CAR	Multiple sclerosis	De Paula Pohl et al., 2020 (185)	
SOD1	CAR	Amyotrophic lateral sclerosis	Graber et al., 2024 (186)	
CD19	CAR	B cell-mediated pathologies	Imura et al., 2020 (187), Doglio et al., 2024 (188)	NCT07473154
CD6	CAR	Graft-vs-host disease, Type 1 diabetes	Salhotra et al., 2025 (189)	NCT05993611, NCT07395050
ENTPD3	CAR	Type 1 diabetes	Pieper et al., 2024 (190)	
IL-23R	CAR	Crohn’s disease	Cui et al., 2025 (191)	
OX40L	CAR	Graft-vs-host disease	Rui et al., 2024 (192)	
Flagellin	CAR	Inflammatory bowel disease	Boardman et al., 2023 (193)	
AAV capsid	CAR	Immunogenic responses to AAV gene therapies	Arjomandnejad et al., 2021 (194)	
FVIII	CAR	Immunogenic responses to FVIII replacement therapy	Yoon et al., 2017 (195)	
FVIII	BAR	Immunogenic responses to FVIII replacement therapy	Zhang et al., 2018 (196)	

Whereas TCR-directed Tregs recapitulate the biological niche of natural counterparts, HLA restriction limits the targetable patient population of a single product. CAR engineering provides an alternative strategy by enabling Treg activation through HLA-independent antigen recognition. An exception to this is CAR-Tregs directed against HLA-A2 (A2), which have demonstrated the feasibility of CAR-mediated allospecific regulation in multiple preclinical studies (134, 182, 183). Clinical evidence from small cohorts of allotransplantation indicate trafficking of CAR-Treg to the A2+ allograft (199) and benefit in tapering of immunosuppressants (200), but the approach is specifically limited to A2- patients receiving an A2+ allograft. CARs targeting non-HLA surface or extracellular antigens offer a more generalizable approach. By recognizing tissue-restricted or inflammation-associated antigens directly, such CAR-Tregs can be preferentially enriched at sites of disease rather than relying on antigen presentation within secondary lymphoid structures. Pieper et al. identified a pancreatic beta islet-specific antigen ENTPD3 suitable for targeting by CAR. Murine surrogate ENTPD3 CAR-Tregs controlled disease in a preclinical model of T1D and enabled enrichment in pancreatic islets compared to irrelevant CAR-Tregs. The group also assessed several candidate CARs targeting the human ENTPD3 antigen to derive a promising lead capable of antigen-dependent suppression and low antigen density response (190). In neuroinflammatory settings, human Tregs engineered with MBP or MOG-specific CARs reduced disease severity In an experimental model of autoimmune encephalomyelitis (185), and CAR-Tregs selectively targeting a mutant SOD1 may be beneficial in ALS (186). CD19-targeted (187, 188) and CD6-targeted (189) CAR-Tregs, though not organ-restricted, exemplify targeted regulation of a defined pathogenic cell population with predictable anatomical distribution (189). Other work has focused on antigens preferentially upregulated during inflammation. For example, CAR-Tregs recognizing citrullinated proteins (CitP) have been developed for rheumatoid arthritis (184). Early clinical results suggest a dose-dependent therapeutic effect, with higher doses producing more consistent and durable reductions in disease activity scores than lower-dose treatment (201). Cui et al. engineered Tregs expressing an IL-23R-specific CAR to suppress intestinal inflammation in Crohn’s disease, where IL-23R upregulation is associated with inflammation (191). A potential biological caveat of CAR-Tregs targeting antigens not found on professional APCs could be the limited ability of CD80/CD86 downregulation by CTLA4 (35, 36), an important Treg suppressive mechanism described above. Tregs expressing a CAR targeting OX40L, a costimulatory ligand preferentially expressed on activated APCs, provided superior control of xenogeneic GvHD than control Tregs and reduced CD80/CD86 on dendritic cells *in vitro* (192). Overall, these studies highlight the potential versatility of CAR-Tregs in regulating immune responses both in a tissue-restricted manner or directly at the inflamed milieu.

Using CARs to target non-self antigens can be applied to indications where immune responses to foreign entities are unwarranted. Boardman et al. developed a CAR-Treg against the bacterial flagellin FliC protein (193), which may limit intestinal immunopathology following microbial breach of the intestinal barrier. To potentially mitigate immune responses against the AAV vector in gene therapies, Arjomandnejad et al. generated CAR-Tregs using a binder that is broadly reactive to AAV serotypes (194). Another example of CAR-Treg for inducing tolerance to a therapeutic is using CARs targeting FVIII (195), which is a non-HLA-restricted iteration of an earlier approach using FVIII-specific TCR (181). Recombinant FVIII is used to treat hemophilia A, but neutralizing immune responses can hinder its efficacy. The same group also developed a B-cell-targeting Antibody Receptor (BAR), where a portion of FVIII is encoded as the extracellular portion of a CAR to engage antigen-specific B cells (196). Both the CAR and BAR-Tregs approaches mitigated anti-FVIII-specific antibody responses (195, 196), although it is unclear if BARs can suppress the full breadth of polyclonal antibodies that bind to epitopes outside of its targeting moiety. The Treg approaches directed by recombinant receptors described here are also summarized in **Table 1**. Together, these studies demonstrate the broad applicability of engineered Tregs to address unmet therapeutic challenges, with each category of targeting approaches presenting their own benefits and risks. Given the plethora of preclinical development advancing toward clinical translation, there are significant opportunities for the field going forward to delineate the optimal Treg antigen-receptor axis for the respective indication.

### Challenges and considerations for translating Treg therapies

The above engineering strategies offer a way forward to better equip Treg cellular drugs for potent efficacy in the clinic. A perspective on the translational considerations pertaining to each of the more established engineering strategies (FOXP3 stabilization, cytokine support, and antigen specificity) is presented here, to supplement extensive coverage on the nonclinical development framework of Treg and T cell therapies found elsewhere (202). First, stabilizing FOXP3 or other Treg-associated factors, as well as delivering therapeutic payloads in general, typically necessitates genetic modifications. These modifications present genotoxicity risks, most concerning of which is the inadvertent transformation of engineered cells. Engineering mature human T cells using lentiviral or retroviral vectors have a long-standing safety record, although rare cases of insertional mutagenesis may have occurred (203). Alternatively, site-specific enzymes can be used for genetic modifications, such as CRISPR/Cas9. While they are more directed than semi-randomly integrating viral vectors, the potential for off-target editing remains (204). With either approach, careful assessment of genome-wide alterations, chromosomal integrity, vector integration profile, and potential for tumorigenesis in the drug product are crucial. Related to the concerns of tumorigenesis, cytokine support engineering may also increase the potential for uncontrolled survival and expansion, which has been previously observed with human Tconv cells (205). Therefore, cytokine signaling engineering needs to consider orthogonality (limited impact on non-engineered cells) and controllability (can be turned off if needed) to balance safety with the desired therapeutic benefit, for example, a constitutive cytokine signaling module may warrant co-expression of a suicide gene for added safety assurance. Regarding antigen receptor engineering, Treg therapies can draw learnings from the Tconv field. Tregs succumb to exhaustion from tonic-signaling CARs (206) similar to Tconv (207), highlighting the need for appropriate CAR design. In contrast to Tconv therapies, the potency derived from recombinant antigen receptors manifest uniquely with Treg biology. Cochrane et al. reported that the supraphysiological signaling of CARs may induce an inflammatory, effector-like program in human Tregs (208), which would be detrimental for a therapy meant to control excessive inflammation. Given this observation, it would be prudent to include Treg stability as a parameter when assessing novel antigen receptors. Assuming Tregs maintain the desired suppressive function, non-localized suppression of beneficial immunity may be a concern when targeting antigens that lack tissue specificity. While the risk-benefit trade-off may be tolerable depending on disease severity, preclinical models of non-selective immune suppression should be implemented where applicable (209). All in all, engineered Treg therapies present added considerations and development complexities compared to unmodified natural Treg products in exchange for potential improvements in efficacy.

## Future perspective - engineering modules for regenerative Treg therapy

Specialized non-lymphoid functions of tissue Tregs that extend beyond their classical immune suppressive roles are becoming increasingly recognized and have been recently highlighted organ-by-organ in seminal reviews (37, 210, 211). Here we examine how these emerging functions may inform next-generation Treg engineering by grouping them into three categories; 1) metabolic control, 2) tissue repair and restoration of parenchymal integrity, and 3) stem-cell niche support and regeneration. By doing so we attempt to tease out unifying mechanistic toggles to reveal druggable pathways or engineering targets that could be used to enhance the disease-correction potential of this modality.

### Metabolic control

Across mammalian tissues, Tregs have emerged as evolutionarily conserved integrators of immune restraint and metabolic fitness in organs with high energetic demand. Beyond controlling inflammation, tissue-adapted Tregs tune parenchymal metabolism, stromal cell behavior, and niche integrity (212, 213) to preserve organ function during physiological stress such as nutrient excess (26, 214), cold exposure (40, 215, 216), and exercise (39, 217).

Tregs with tissue-specific transcriptional programs, later coined Tissue Tregs, were first described in the VAT depot (218, 219). These cells deploy PPARγ-driven transcriptional circuits that encode chemokine receptors and lipid metabolism proteins facilitating their tissue accumulation, survival and function within lipid-rich environments (219, 220). The PPARγ program is reinforced by stromal-derived IL-33 acting through the receptor ST2 (IL1RL1) (221). Loss of VAT Tregs promotes adipose inflammation, insulin resistance, and hyperglycemia (16, 218, 222–224), while restoring their abundance via IL-2/IL-2 complexes or exogenous IL-33 reverses diabetogenic phenotypes in experimental models (16, 221). Beyond their immunoregulatory functions, ST2^+^ VAT Tregs directly regulate the adipose niche through the production of oncostatin M (OSM), which acts via a conserved stromal OSMR pathway to restrain aberrant adipogenesis, limit inflammation, and maintain insulin sensitivity (225). These data demonstrate a direct role for Tregs in regulating local progenitor cell function and suggest that tissue Treg tuning may be more effective than strategies focused solely on singular inflammatory cytokines or circulating Treg numbers. Consistent with this, the exercise-induced hormone irisin promotes stromal IL-33 production and preserves ST2^+^ VAT Tregs, reducing inflammation and improving insulin sensitivity (226). Targeting this or similar Treg niche supporting mechanisms may be a novel approach to address the persistent immune dysfunction underlying “obesity memory” and the incomplete metabolic resetting observed after weight loss (227), attributed in-part to Treg-dysfunction (228) and de-specialization (229). Recent studies have added to our understanding of the heterogenous nature of the VAT Treg compartment, also identifying Tbet^+^ CXCR3^+^ subset as important for glucose homeostasis in female mice (223, 230), revealing additional molecular checkpoints, that may be exploited for more precise and patient-specific therapeutic targeting in obesity and metabolic disease.

Unlike white adipose tissue depots, brown adipose tissue (BAT) dissipates energy via UCP1-mediated thermogenesis. Here BAT-resident Tregs support adaptive energy expenditure, and thermogenic fitness by restraining local type-1 inflammatory signals (40, 215, 216). Moreover, the IL-18R1^+^ Treg subset acts as a metabolic sentinel during cold stress, as Treg-specific loss of IL-18R1 unleashes IFN-γ mediated mitochondrial dysfunction and impaired thermogenic fitness (40). Notably, IL-18R1^+^ BAT-Tregs display a distinct transcriptomic profile characterized by high expression of CD73, suggesting that purinergic signaling may contribute to thermogenic regulation in addition to immune control (231, 232). Additionally, ST2^+^ Tregs are maintained within the BAT niche via leptin-induced IL-33 production by perineurial cells that adorn sympathetic neuronal axon bundles, and their absence abrogates metabolic adaptation to fasting (214). Together, these data demonstrate a poly-functional Treg compartment that integrate distinct alarmin signals to coordinate tissue metabolism. IL-18-associated programs support thermogenic output and mitochondrial fitness during cold stress, whereas IL-33-driven programs support adaptation during nutrient stress.

A parallel paradigm operates in skeletal muscle, where exercise promotes the expansion and functional maturation of muscle-resident Tregs through myofiber derived IL-6 and stromal derived IL-33 that maintain a tissue Treg phenotype characterized by ST2 and Areg expression (17, 39). Among these cues, IL-6 appears particularly important, as knockout of IL-6 receptor leads to an absence of Treg expansion in response to exercise and subsequent muscle weakness and loss (39). These effects are associated with a loss of local IFN-γ control, mitochondrial dysfunction and oxidative phosphorylation capacity within myofibrils (217). Thus, similar to the IL-18R1 and ST2 pathways described in the adipose, the IL-6R axis may represent a candidate module for engineering Tregs with enhanced fitness in metabolic tissues.

Together, these vignettes illustrate how tissue Tregs integrate environmental and alarmin-derived cues to adopt specialized programs that couple immune suppression with direct regulation of tissue metabolism. These pathways may provide modules for future pharmacologic or cell-engineering approaches aimed at generating Tregs capable of adapting to unique metabolic niches and correcting metabolic imbalances to restore tissue function in disease and aging.

### Tissue repair

Studies over the last decade have established that Tregs are not passive bystanders of repair, but important tissue sentinels that detect injury and coordinate reparative responses. Tissue-resident Tregs express receptors, ST2 and IL-18R1, that sense the release of damage and inflammatory inducible alarmins IL-33 and IL-18, respectively. These signals induce a tissue Treg repair module, including upregulation to salient repair factor Areg, independent to TCR engagement (233). Areg acts directly on epithelial and parenchymal progenitors via epidermal growth factor receptor (EGFR) to preserve barrier integrity, promote proliferation, and enhance survival. This mechanism is conserved across tissues, including skeletal muscle (17), lung (234) and heart (235), where Treg-specific deletion impairs regeneration without exacerbating inflammation. Emerging evidence suggests a similar role for Areg-producing Tregs in skin wound healing (236) and in the central nervous system (CNS), where Areg limits astrogliosis, supports neural recovery (237), and staves off depression (238). Interestingly, the Areg-repair mechanism may be self-reinforcing in a paracrine and non-paracrine manner (236, 239).

Beyond the direct epithelial modifying effects of Areg, tissue Tregs also exert repair via the production of Th-2 cytokines, particularly IL-10, IL-4 and IL-13, which suppress Th-1 type inflammation, and reshape myeloid responses toward tissue healing. In the lung, IL-33-induced IL-13 production supports lung recovery following acute injury (240), whereas Notch4 signaling antagonizes tissue-repair programs by suppressing IL-18-induced Areg expression (241). Similar IL-33-dependent pathways operate in skeletal (242–244) and cardiac muscle (245–248), where Tregs regulate macrophage differentiation and tissue remodeling. Additional tissue-specific reparative mechanisms have also emerged, including Treg-derived osteopontin, which direct CNS microglia to engage a reparative program that promotes oligodendrogenesis and white matter repair (249).

While niche specific factors can differ between organs, conserved pathways of repair emerge in which Tregs integrate damage-associated signals and reshape local stromal and myeloid responses to favor repair over chronic inflammation. Recurring nodes include IL-33/ST2, IL-18/IL-18R1, Areg/EGFR, and osteopontin-dependent pathways. Therapeutically manipulating these pathways may enable precision tuning of endogenous repair responses.

### Sustaining the stem cell niche and regenerative potential

An exciting insight into the potential of Tregs for regenerative healing was highlighted by Hui et al (250, 251) when studying Foxp3-expressing T cells in zebrafish (zTregs). zTregs infiltrated multiple organs upon injury, such as the heart, retina, and spinal cord where they secreted tissue-specific factors that promoted progenitor proliferation. Transcriptomic and functional analyses identified key modulated pathways, including IGF signaling (igf2a) for cardiomyocyte regeneration, CNTF and FGF family ligands for retinal repair, and neurotrophin signaling (e.g., nrg1) for spinal cord recovery. Ablation of zTregs selectively impaired precursor expansion and structural recovery, while exogenous delivery of each key factor rescued regeneration. These data highlight the evolutionary conservation of Foxp3, and tissue-specific functions, raising the compelling possibility that mammalian Tregs can be enabled, reprogrammed or engineered to access analogous pathways for therapeutic modulation.

A tissue-regenerative role for Tregs in mammalian organisms is emerging. One of the best-characterized examples is in the skin, where Notch ligand Jagged-1^+^ (Jag1) Tregs localize to the hair follicle (HF) bulge, and directly regulate hair follicle stem cell (HFSC) activity through Jag1-Notch signaling, promoting HFSC activation and hair growth (18, 252). HF bulge Tregs also directly support HFSC differentiation during depilation through the production of TGF-β3 via SMAD2/3 signaling (253, 254). Together, these findings identify Jag1-Notch and TGF-β3-SMAD pathways as direct mechanisms by which Tregs regulate HFSC behavior, shielded from inflammatory perturbations via enriched expression of the high-affinity IL-2 receptor (255). Consistent with these observations, Notch signaling has been shown to be critical for the maintenance of stem cell renewal across multiple adult tissues (256–258), while the integration of Notch and SMAD2/3 signaling determines stem cells fate decisions, by balancing self-renewal versus differentiation (259–262). A stem-cell niche supporting role for Tregs has also emerged in protected tissues such as the bone-marrow and CNS. In the bone-marrow, Tregs protect hematopoietic stem cells from inflammatory attrition via IL-10-dependent circuits and purinergic signaling (263). In parallel, bone-resident Tregs reinforce a regenerative microenvironment via CCL1/CCR8 recruitment and secretion of progranulin that promotes osteogenic differentiation of skeletal stem cells into osteoblasts responsible for new bone formation (264). Similarly in the CNS, Tregs limit-inflammation to preserve stem cells function important for neurogenic capacity and myelination (237, 265), and also exhibit direct effects via Treg-derived IL-10 (266) and CCN3 (267).

Collectively, these studies reveal that Tregs can directly regulate stem cell function, progenitor-cell fate and regenerative capacity across multiple tissues in animal models of human disease. While the degree to which these programs can be therapeutically harnessed in humans remains incompletely defined, such approaches would extend Treg-based medicine beyond immune regulation toward active restoration of tissue structure and function.

The above-described non-canonical roles of Tregs, spanning metabolic regulation, tissue repair, and regeneration, have largely been discovered and extensively characterized in animal models, but appear to be governed by ‘tissue-programmed’ transcriptional signatures that are replicated in human Tregs (268). This conservation opens the possibility of translating non-canonical tissue Treg functions through pharmacologic programming or genetic engineering approaches. Such strategies could generate tissue-adapted Tregs equipped with defined reparative, metabolic, or regenerative programs. These engineered cells would be expected to home to diverse tissue sites (269, 270), and integrate local cues to coordinate both immune regulation, and tissue-specific functions. These new discoveries broaden the therapeutic implications of Tregs, and identify a growing repertoire of druggable or genetically-modifiable toggles that can be used to extend the efficacy of immune-suppressive strategies (addressed below). Such a regenerative approach would have large impacts in multiple fields of medicine, including, but not limited to, organ transplantation, neurodegeneration, and metabolic disorders (**Figure 3**).

**Figure 3:**
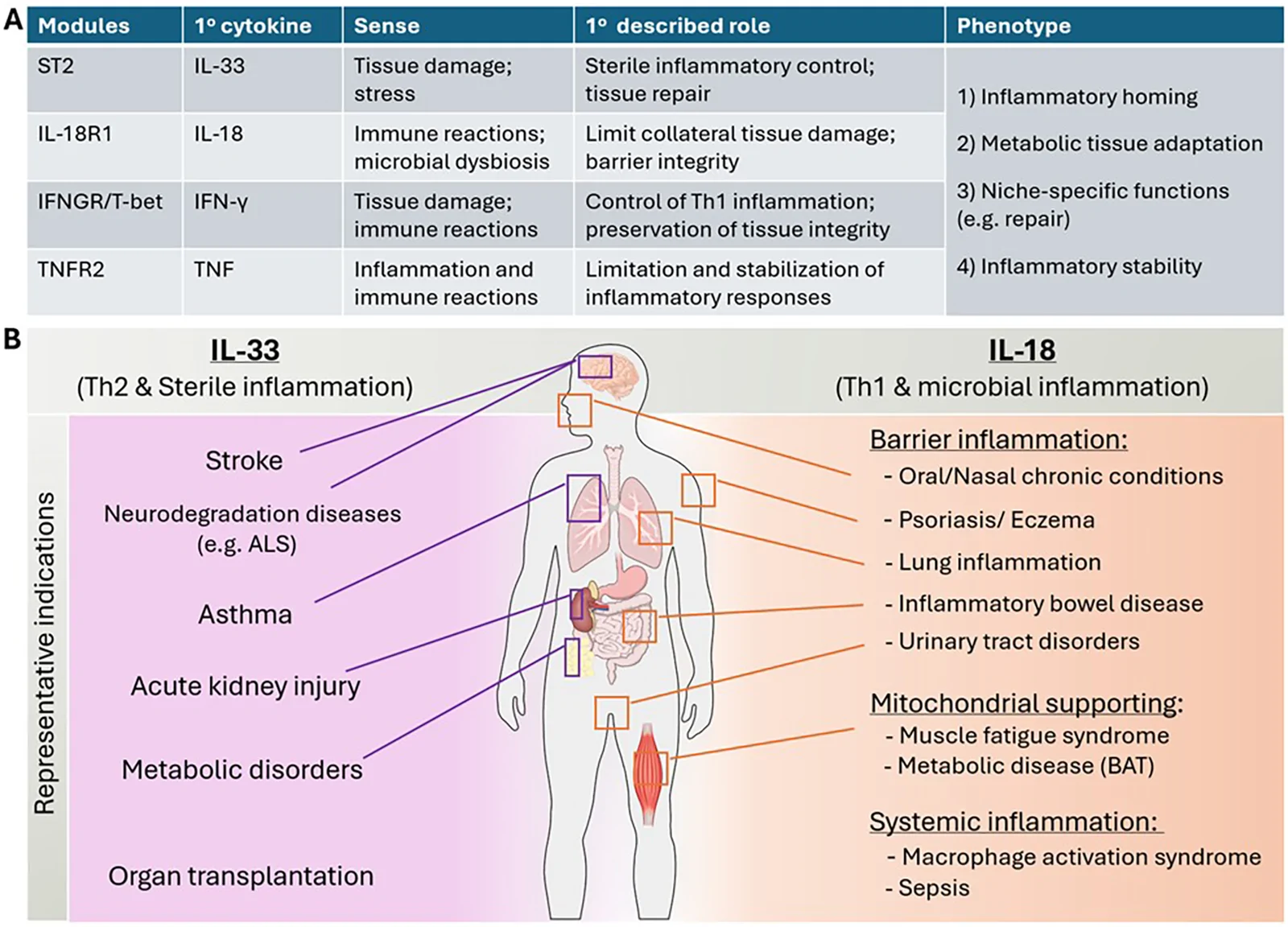
Tissue Tregs, shaped by cytokines, are positioned as effective combatants to target a variety of inflammatory conditions. **(A)** Table describing examples of key ‘tissue’ Treg transcriptional modules. Cytokine source and cognate receptor expression dictate what each factor senses and their primary function in the body. Each tissue Treg model elicits a niche specific phenotype. **(B)** Examples of inflammatory conditions best suited for intervention by indicated alarmin-tuned Tregs (IL-33 vs IL-18).

### Unlocking Treg phenotypes and functions for target tissue and disease-tailored activity

The non-canonical metabolic, reparative, and regenerative programs described above identify a new class of engineering opportunities for Treg-based therapies. Whereas most current Treg engineering strategies focus on improving stability, persistence, or antigen specificity, emerging approaches seek to deliberately induce tissue-adapted phenotypes capable of supporting tissue repair and restoration of organ function. The signaling nodes highlighted in the preceding sections, including IL-33/ST2, IL-18/IL-18R1, AREG/EGFR, TNF/TNFR2, IL-6/IL-6R, and Jag1/Notch, represent some of the first candidate modules that may be exploited to generate tissue-specialized Treg products. However, compared with the advancements achieved for stability and specificity engineering, strategies designed to impart non-canonical functions remain nascent.

A notable early strategy to enhance tissue-supportive, homing and stability phenotypes has been to manipulate the IL-33-ST2 pathway. IL-33 is an alarmin released by injured tissue, with a role in eliciting tissue repair functions in ST2-expressing Tregs (37, 233, 271). Pioneer studies first targeted this module through *in vivo* delivery of cytokine and cytokine-derived biologics. Stremska et al (272) described a hybrid cytokine (IL233) that exhibits activities of both IL-2 and IL-33 and was shown to proportionally increase renal homing of Tregs following ischemic reperfusion injury compared to non-treated mice. In addition, adoptively transferred Tregs from IL233 treated animals better protected recipients from nephrotoxicity. Similarly, adoptive transfer of Tregs treated with IL-33 exhibited enhanced protection of a skin allograft, in part due to more rapid homing to the graft site (273). Interestingly, injection of an IL-2 mutein was found to promote antigen-specific heart and kidney allograft acceptance in mice via expansion of ST2^+^ Tregs with enhanced suppressive function (274).

Together, these data lend support to the idea that enhanced tissue emigration and function can be achieved via *in vivo* cytokine reprogramming. However, similar to exogenous delivery of IL-2, this strategy is not entirely Treg specific (also targeting ILC2s); may be oscillatory in nature and thus less durable in the autoimmune context (275); and ex-vivo approaches are limited by the fact that human tissue Tregs are comparatively rare, with easily accessible human Tregs circulating in the blood typically expressing low levels of ST2, also potentially limiting the success of *in vivo* expansion of this subset, as observed in mice (276). To overcome these limitations, Lam et al., sought to impart the tissue-repair program to isolated circulating Tregs, through ectopic overexpression of ST2. ST2-transduced human Tregs produced higher levels of amphiregulin, a repair-focused EGFR ligand, in an IL-33-dependent manner, whereas control vector Tregs did not respond to IL-33. Notably, IL-33 treatment of these ST2^+^ Tregs did not appreciably impact their suppressive effect, suggesting that the ectopic IL-33-ST2 axis does not simply amplify all Treg functions but may specifically boost the pro-repair capacity (277). These data provide proof-of-concept that tissue-supportive programs can be engineered independently of canonical suppressive activity, raising the possibility of generating Treg products with customized reparative modules tailored to specific disease settings.

The above examples describe cytokine and genetic engineering approaches to modulate the IL-33-ST2-AREG axis, providing support for pursuing additionally identified pathways to enhance tissue-specialized Treg functions. Indeed, tissue resident Tregs are characterized by higher levels of TNFR2, and costimulation via this pathway enforces a non-lymphoid tissue phenotype in human Tregs comprising tissue regeneration gene sets (278). Signaling through this pathway appears critical for the stability, metabolic fitness, proliferative, and suppressive capacity of Tregs within inflammatory tissues (279–282), and is thus important for protection from both inflammatory disease and GvHD (45, 283–285). Trials utilizing a TNFR2 agonist to treat atopic dermatitis are underway and will provide further insight into the utility and tissue-restorative capacity of TNFR2 targeting (NCT06934252).

Emerging evidence highlights tissue Tregs to be a highly heterogeneous cell population with distinct transcriptomes and context-specific activities (286), imparted by a complex combinatorial web of transcription factor activities (287). Transcriptomic mapping of human tissue resident Tregs under health and disease offers a powerful approach to identify both novel tissue-programming factors and druggable pathways beyond those already highlighted, including L-33/ST2, IL-18/IL-18R1, OSM/OSMR, TNF/TNFR2, Jag1/Notch and TGF-β3/SMAD. Such programs may be harnessed through a spectrum of interventions ranging from culture-based conditioning and cytokine-guided differentiation to receptor engineering and targeted genome modification. Collectively, these early studies provide proof-of-concept that non-canonical Treg programs can be enhanced through cytokine delivery, receptor engineering, and ex vivo programming strategies. As our understanding of tissue-Treg signaling networks continues to improve, future efforts may increasingly shift from engineering broadly suppressive cells toward engineering tissue-specific metabolic, reparative, and regenerative functions tailored to individual disease contexts. The challenge ahead will be to identify tissue-Treg modules that are both mechanistically important and sufficiently tractable for translation into clinically manufacturable cell therapies.

## Summary

Over the past decade, our understanding of regulatory T cells has expanded from viewing Tregs as a relatively uniform suppressive population to a highly diversified, tissue-adapted network of immune regulators. This period has yielded transformative insights into Treg developmental ontogeny, transcriptional and epigenetic wiring, cytokine dependencies, and the profound influence of local environmental cues on their phenotype and function. These discoveries have redefined Tregs as multifunctional cells capable not only of suppressing pathological immunity but also of orchestrating tissue repair, supporting stem-cell niches, and modulating metabolic homeostasis across organ systems. Such broad biological capability that integrates immune restraint with restoration of tissue function has laid a mechanistic foundation for therapies that aim not merely to dampen inflammation, but to re-establish durable homeostasis at diseased tissue sites.

As Treg-based therapies advance toward clinical maturity, the field faces a new set of challenges that require equally sophisticated solutions. Chief among these is the need to enhance the *precision*, *persistence*, and contextual *fitness* of adoptively transferred Tregs in environments where inflammatory stress, cytokine deprivation, and metabolic instability threaten their lineage integrity. Future progress will require deeper mechanistic understanding of the molecular toggles governing Treg stability (FOXP3 maintenance, cytokine sensing, chromatin locking), tissue-specific functional modules (TNF/TNFR2, IL-33/ST2 and IL-18/IL-18R1 circuits, PPARγ-directed metabolic programs), and the migratory and residency pathways that determine where Tregs act and for how long. These insights will be critical not only for refining adoptive Treg therapies but also for guiding the development of new classes of engineered Tregs that can withstand inflammatory pressure, execute context-specific functions, and predictably integrate into diseased tissues. Equally important will be the development of strategies that couple antigen specificity with tissue programming, ensuring that engineered Tregs engage the right targets, in the right microenvironments, with the right effector modules.

Within this integrated vision, the optimal Treg therapeutic is not simply a suppressor cell but a programmable, tissue-tuned immune sentinel, capable of homing to diseased sites, resisting destabilization, enforcing antigen-specific tolerance, and actively repairing damaged tissues. Such multifunctional cellular medicines hold the potential to fundamentally reshape treatment paradigms for autoimmune, inflammatory, metabolic, and degenerative diseases.

## References

[1] Sakaguchi S Yamaguchi T Nomura T Ono M . Regulatory T cells and immune tolerance. Cell. (2008) 133:775–87. doi: 10.1016/j.cell.2008.05.009 18510923

[2] Fontenot JD Gavin MA Rudensky AY . Pillars article: Foxp3 programs the development and function of CD4+CD25+ regulatory T cells. Nat. Immunol. 2003. 4: 330-336. J Immunol. (2017) 198:986–92. doi: 10.1038/ni904 28115587

[3] Hori S Nomura T Sakaguchi S . Pillars article: Control of regulatory T cell development by the transcription factor Foxp3. Science 2003. 299: 1057-1061. J Immunol. (2017) 198:981–5. doi: 10.1093/jimmunol/198.3.981 28115586

[4] Khattri R Cox T Yasayko SA Ramsdell F . Pillars article: An essential role for Scurfin in CD4+CD25+ T regulatory cells. Nat. Immunol. 2003. 4: 337-342. J Immunol. (2017) 198:993–8. doi: 10.1038/ni909 28115588

[5] Brunkow ME Jeffery EW Hjerrild KA Paeper B Clark LB Yasayko SA . Disruption of a new forkhead/winged-helix protein, scurfin, results in the fatal lymphoproliferative disorder of the scurfy mouse. Nat Genet. (2001) 27:68–73. doi: 10.1038/83784 11138001

[6] Bennett CL Christie J Ramsdell F Brunkow ME Ferguson PJ Whitesell L . The immune dysregulation, polyendocrinopathy, enteropathy, X-linked syndrome (IPEX) is caused by mutations of FOXP3. Nat Genet. (2001) 27:20–1. doi: 10.1038/83713 11137993

[7] Lio CW Hsieh CS . Becoming self-aware: the thymic education of regulatory T cells. Curr Opin Immunol. (2011) 23:213–9. doi: 10.1016/j.coi.2010.11.010 21146972 PMC3061250

[8] Yang S Fujikado N Kolodin D Benoist C Mathis D . Immune tolerance. Regulatory T cells generated early in life play a distinct role in maintaining self-tolerance. Science. (2015) 348:589–94. doi: 10.1126/science.aaa7017 25791085 PMC4710357

[9] Jordan MS Boesteanu A Reed AJ Petrone AL Holenbeck AE Lerman MA . Thymic selection of CD4+CD25+ regulatory T cells induced by an agonist self-peptide. Nat Immunol. (2001) 2:301–6. doi: 10.1038/86302 11276200

[10] Apostolou I Sarukhan A Klein L von Boehmer H . Origin of regulatory T cells with known specificity for antigen. Nat Immunol. (2002) 3:756–63. doi: 10.1038/ni816 12089509

[11] Malchow S Leventhal DS Nishi S Fischer BI Shen L Paner GP . Aire-dependent thymic development of tumor-associated regulatory T cells. Science. (2013) 339:1219–24. doi: 10.1126/science.1233913 23471412 PMC3622085

[12] Dikiy S Ghelani AP Levine AG Martis S Giovanelli P Wang ZM . Terminal differentiation and persistence of effector regulatory T cells essential for preventing intestinal inflammation. Nat Immunol. (2025) 26:444–58. doi: 10.1038/s41590-024-02075-6 39905200 PMC11876075

[13] Liston A Rudensky AY . Thymic development and peripheral homeostasis of regulatory T cells. Curr Opin Immunol. (2007) 19:176–85. doi: 10.1016/j.coi.2007.02.005 17306520

[14] Malchow S Leventhal DS Lee V Nishi S Socci ND Savage PA . Aire enforces immune tolerance by directing autoreactive T cells into the regulatory T cell lineage. Immunity. (2016) 44:1102–13. doi: 10.1016/j.immuni.2016.02.009 27130899 PMC4871732

[15] Aschenbrenner K D'Cruz LM Vollmann EH Hinterberger M Emmerich J Swee LK . Selection of Foxp3+ regulatory T cells specific for self antigen expressed and presented by Aire+ medullary thymic epithelial cells. Nat Immunol. (2007) 8:351–8. doi: 10.1038/ni1444 17322887

[16] Kolodin D van Panhuys N Li C Magnuson AM Cipolletta D Miller CM . Antigen- and cytokine-driven accumulation of regulatory T cells in visceral adipose tissue of lean mice. Cell Metab. (2015) 21:543–57. doi: 10.1016/j.cmet.2015.03.005 25863247 PMC4747251

[17] Burzyn D Kuswanto W Kolodin D Shadrach JL Cerletti M Jang Y . A special population of regulatory T cells potentiates muscle repair. Cell. (2013) 155:1282–95. doi: 10.1016/j.cell.2013.10.054 24315098 PMC3894749

[18] Ali N Zirak B Rodriguez RS Pauli ML Truong HA Lai K . Regulatory T cells in skin facilitate epithelial stem cell differentiation. Cell. (2017) 169:1119–29:e1111. doi: 10.1016/j.cell.2017.05.002 28552347 PMC5504703

[19] Shiow LR Rosen DB Brdickova N Xu Y An J Lanier LL . CD69 acts downstream of interferon-alpha/beta to inhibit S1P1 and lymphocyte egress from lymphoid organs. Nature. (2006) 440:540–4. doi: 10.1038/nature04606 16525420

[20] Bankovich AJ Shiow LR Cyster JG . CD69 suppresses sphingosine 1-phosophate receptor-1 (S1P1) function through interaction with membrane helix 4. J Biol Chem. (2010) 285:22328–37. doi: 10.1074/jbc.M110.123299 20463015 PMC2903414

[21] Malhotra N Leyva-Castillo JM Jadhav U Barreiro O Kam C O'Neill NK . RORalpha-expressing T regulatory cells restrain allergic skin inflammation. Sci Immunol. (2018) 3:eaao6923. doi: 10.1126/sciimmunol.aao6923 29500225 PMC5912895

[22] Wohlfert EA Grainger JR Bouladoux N Konkel JE Oldenhove G Ribeiro CH . GATA3 controls Foxp3(+) regulatory T cell fate during inflammation in mice. J Clin Invest. (2011) 121:4503–15. doi: 10.1172/JCI57456 21965331 PMC3204837

[23] Floess S Freyer J Siewert C Baron U Olek S Polansky J . Epigenetic control of the foxp3 locus in regulatory T cells. PloS Biol. (2007) 5:e38. doi: 10.1371/journal.pbio.0050038 17298177 PMC1783672

[24] Huehn J Polansky JK Hamann A . Epigenetic control of FOXP3 expression: the key to a stable regulatory T-cell lineage? Nat Rev Immunol. (2009) 9:83–9. doi: 10.1038/nri2474 19114986

[25] Zheng Y Josefowicz S Chaudhry A Peng XP Forbush K Rudensky AY . Role of conserved non-coding DNA elements in the Foxp3 gene in regulatory T-cell fate. Nature. (2010) 463:808–12. doi: 10.1038/nature08750 20072126 PMC2884187

[26] Li C Spallanzani RG Mathis D . Visceral adipose tissue Tregs and the cells that nurture them. Immunol Rev. (2020) 295:114–25. doi: 10.1111/imr.12850 32162339

[27] van der Veeken J Campbell C Pritykin Y Schizas M Verter J Hu W . Genetic tracing reveals transcription factor Foxp3-dependent and Foxp3-independent functionality of peripherally induced Treg cells. Immunity. (2022) 55:1173–84:e1177. doi: 10.1016/j.immuni.2022.05.010 35700740 PMC9885886

[28] Dikiy S Rudensky AY . Principles of regulatory T cell function. Immunity. (2023) 56:240–55. doi: 10.1016/j.immuni.2023.01.004 36792571

[29] Vignali DA Collison LW Workman CJ . How regulatory T cells work. Nat Rev Immunol. (2008) 8:523–32. doi: 10.1038/nri2343 18566595 PMC2665249

[30] Delacher M Imbusch CD Hotz-Wagenblatt A Mallm JP Bauer K Simon M . Precursors for nonlymphoid-tissue Treg cells reside in secondary lymphoid organs and are programmed by the transcription factor BATF. Immunity. (2020) 52:295–312:e211. doi: 10.1016/j.immuni.2019.12.002 31924477 PMC7026712

[31] Shiohara T Mizukawa Y Hayakawa J Hayakawa K . T-cell dynamics of inflammatory skin diseases. Expert Rev Clin Immunol. (2005) 1:357–68. doi: 10.1586/1744666X.1.3.357 20476987

[32] Iwata M Hirakiyama A Eshima Y Kagechika H Kato C Song SY . Retinoic acid imprints gut-homing specificity on T cells. Immunity. (2004) 21:527–38. doi: 10.1016/j.immuni.2004.08.011 15485630

[33] Pandiyan P Zheng L Ishihara S Reed J Lenardo MJ . CD4+CD25+Foxp3+ regulatory T cells induce cytokine deprivation-mediated apoptosis of effector CD4+ T cells. Nat Immunol. (2007) 8:1353–62. doi: 10.1038/ni1536 17982458

[34] Scheffold A Huhn J Hofer T . Regulation of CD4+CD25+ regulatory T cell activity: it takes (IL-)two to tango. Eur J Immunol. (2005) 35:1336–41. doi: 10.1002/eji.200425887 15827965

[35] Wing K Onishi Y Prieto-Martin P Yamaguchi T Miyara M Fehervari Z . CTLA-4 control over Foxp3+ regulatory T cell function. Science. (2008) 322:271–5. doi: 10.1126/science.1160062 18845758

[36] Qureshi OS Zheng Y Nakamura K Attridge K Manzotti C Schmidt EM . Trans-endocytosis of CD80 and CD86: a molecular basis for the cell-extrinsic function of CTLA-4. Science. (2011) 332:600–3. doi: 10.1126/science.1202947 21474713 PMC3198051

[37] Munoz-Rojas AR Mathis D . Tissue regulatory T cells: regulatory chameleons. Nat Rev Immunol. (2021) 21:597–611. doi: 10.1038/s41577-021-00519-w 33772242 PMC8403160

[38] Yan Y Ramanan D Rozenberg M McGovern K Rastelli D Vijaykumar B . Interleukin-6 produced by enteric neurons regulates the number and phenotype of microbe-responsive regulatory T cells in the gut. Immunity. (2021) 54:499–513:e495. doi: 10.1016/j.immuni.2021.02.002 33691135 PMC8133394

[39] Becker M Joseph SS Garcia-Carrizo F Tom RZ Opaleva D Serr I . Regulatory T cells require IL6 receptor alpha signaling to control skeletal muscle function and regeneration. Cell Metab. (2023) 35:1736–51:e1737. doi: 10.1016/j.cmet.2023.08.010 37734370 PMC10563138

[40] Zammit NW Vargas-Castillo A Langston PK Wang G Zhou Y Spiegelman BM . Regulatory T cells in brown adipose tissue safeguard thermogenesis by restraining interferon-gamma-producing lymphocytes. Sci Immunol. (2025) 10:eads0478. doi: 10.1126/sciimmunol.ads0478 40712046 PMC12363373

[41] Harrison OJ Srinivasan N Pott J Schiering C Krausgruber T Ilott NE . Epithelial-derived IL-18 regulates Th17 cell differentiation and Foxp3(+) Treg cell function in the intestine. Mucosal Immunol. (2015) 8:1226–36. doi: 10.1038/mi.2015.13 25736457 PMC4368110

[42] Alvarez F Istomine R Da Silva Lira Filho A Al-Aubodah TA Huang D Okde R . IL-18 is required for the T(H)1-adaptation of T(REG) cells and the selective suppression of T(H)17 responses in acute and chronic infections. Mucosal Immunol. (2023) 16:462–75. doi: 10.1016/j.mucimm.2023.05.004 37182738

[43] Yang S Xie C Chen Y Wang J Chen X Lu Z . Differential roles of TNFalpha-TNFR1 and TNFalpha-TNFR2 in the differentiation and function of CD4(+)Foxp3(+) induced Treg cells *in vitro* and *in vivo* periphery in autoimmune diseases. Cell Death Dis. (2019) 10:27. doi: 10.1038/s41419-018-1266-6 30631042 PMC6328545

[44] Chen X Subleski JJ Hamano R Howard OM Wiltrout RH Oppenheim JJ . Co-expression of TNFR2 and CD25 identifies more of the functional CD4+FOXP3+ regulatory T cells in human peripheral blood. Eur J Immunol. (2010) 40:1099–106. doi: 10.1002/eji.200940022 20127680 PMC3096013

[45] Atretkhany KN Mufazalov IA Dunst J Kuchmiy A Gogoleva VS Andruszewski D . Intrinsic TNFR2 signaling in T regulatory cells provides protection in CNS autoimmunity. Proc Natl Acad Sci USA. (2018) 115:13051–6. doi: 10.1073/pnas.1807499115 30498033 PMC6304938

[46] Li H Zeng H . Current status of Treg therapy in transplantation and autoimmune disease. Immunology. (2026) 179(1):1–18. doi: 10.1111/imm.70151 42159047 PMC13432033

[47] Bluestone JA Levings MKK Ramsdell FJ Rudensky AY Tang Q Trzonkowski P . Regulatory T cells: master orchestrators of immune tolerance and tissue homeostasis. Front Sci. (2026) 4. doi: 10.3389/fsci.2026.1792210

[48] Sato Y Passerini L Piening BD Uyeda MJ Goodwin M Gregori S . Human-engineered Treg-like cells suppress FOXP3-deficient T cells but preserve adaptive immune responses *in vivo*. Clin Transl Immunol. (2020) 9:e1214. doi: 10.1002/cti2.1214 33304583 PMC7688376

[49] Meyer EH Salhotra A Gandhi AP Pantin J Patel SS Hoeg RT . Orca-T vs allogeneic hematopoietic stem cell transplantation (Precision-T): a multicenter, randomized phase 3 trial. Blood. (2026) 147:1168–77. doi: 10.1182/blood.2025031313 41385341 PMC13123417

[50] Pidala JA Cieri N Schell MJ Song X Shao Y Cao B . Expanded antigen-specific donor regulatory T cells for GVHD prevention. Blood. (2026). doi: 10.1182/blood.2025030663 42322116 PMC13642963

[51] Whangbo JS Nikiforow S Kim HT Wahl J Reynolds CG Rai SC . A phase 1 study of donor regulatory T-cell infusion plus low-dose interleukin-2 for steroid-refractory chronic graft-vs-host disease. Blood Adv. (2022) 6:5786–96. doi: 10.1182/bloodadvances.2021006625 35475885 PMC9647832

[52] Chandran S Tang Q Sarwal M Laszik ZG Putnam AL Lee K . Polyclonal regulatory T cell therapy for control of inflammation in kidney transplants. Am J Transplant. (2017) 17:2945–54. doi: 10.1111/ajt.14415 28675676 PMC5662482

[53] McCallion O Cross AR Brook MO Hennessy C Ferreira R Trzupek D . Regulatory T cell therapy is associated with distinct immune regulatory lymphocytic infiltrates in kidney transplants. Med. (2025) 6:100561. doi: 10.1016/j.medj.2024.11.014 39731908 PMC7618234

[54] Chandran S Leung JC Vu A Lee K Fitch M Esensten JH . Pharmacokinetics, lineage identity, and trafficking of ex vivo expanded polyclonal regulatory T cells in a prospective randomized clinical trial of kidney transplant recipients with allograft inflammation. Am J Transplant. (2026) 26:744–59. doi: 10.1016/j.ajt.2025.11.011 41314617 PMC12971219

[55] Guinan EC Contreras-Ruiz L Crisalli K Rickert C Rosales I Makar R . Donor antigen-specific regulatory T cell administration to recipients of live donor kidneys: A ONE Study consortium pilot trial. Am J Transplant. (2023) 23:1872–81. doi: 10.1016/j.ajt.2023.06.012 37422112

[56] Thonhoff JR Beers DR Zhao W Pleitez M Simpson EP Berry JD . Expanded autologous regulatory T-lymphocyte infusions in ALS: A phase I, first-in-human study. Neurol Neuroimmunol Neuroinflamm. (2018) 5:e465. doi: 10.1212/NXI.0000000000000465 29845093 PMC5961523

[57] Thonhoff JR Berry JD Macklin EA Beers DR Mendoza PA Zhao W . Combined regulatory T-lymphocyte and IL-2 treatment is safe, tolerable, and biologically active for 1 year in persons with amyotrophic lateral sclerosis. Neurol Neuroimmunol Neuroinflamm. (2022) 9:e200019. doi: 10.1212/NXI.0000000000200019 36038262 PMC9423710

[58] Shneider NA Nesta AV Rifai OM Yasek J Elyaman W Aziz-Zaman S . Clinical safety and preliminary efficacy of regulatory T cells for ALS. NEJM Evid. (2025) 4:EVIDoa2400249. doi: 10.1056/EVIDoa2400249 40261116

[59] Beers DR Zhao W Wang J Zhang X Wen S Neal D . ALS patients' regulatory T lymphocytes are dysfunctional, and correlate with disease progression rate and severity. JCI Insight. (2017) 2:e89530. doi: 10.1172/jci.insight.89530 28289705 PMC5333967

[60] Dong S Hiam-Galvez KJ Mowery CT Herold KC Gitelman SE Esensten JH . The effect of low-dose IL-2 and Treg adoptive cell therapy in patients with type 1 diabetes. JCI Insight. (2021) 6:e147474. doi: 10.1172/jci.insight.147474 34324441 PMC8492314

[61] Bandura M Piotrowska-Mieczkowska M Hennig M Gliwinski M Woloszyn-Durkiewicz A Schoenhoff J . Safety and efficacy of the therapy with CD4 + CD25highCD127-T regulatory cells: When paediatric patient becomes adult. Diabetes Obes Metab. (2026) 28:1392–402. doi: 10.1111/dom.70330 41292422 PMC12803574

[62] Zielinski M Zalinska M Iwaszkiewicz-Grzes D Gliwinski M Hennig M Jazwinska-Curyllo A . Combined therapy with CD4(+) CD25highCD127(-) T regulatory cells and anti-CD20 antibody in recent-onset type 1 diabetes is superior to monotherapy: Randomized phase I/II trial. Diabetes Obes Metab. (2022) 24:1534–43. doi: 10.1111/dom.14723 35441440

[63] Raffin C Vo LT Bluestone JA . Treg cell-based therapies: challenges and perspectives. Nat Rev Immunol. (2020) 20(3):158–172. doi: 10.1038/s41577-019-0232-6 PMC7814338 31811270

[64] Bluestone JA McKenzie BS Beilke J Ramsdell F . Opportunities for Treg cell therapy for the treatment of human disease. Front Immunol. (2023) 14:1166135. doi: 10.3389/fimmu.2023.1166135 37153574 PMC10154599

[65] Cheru NT Osayame Y Sumida TS . Breaking tolerance: an update of Treg dysfunction in autoimmunity. Trends Immunol. (2025) 46:611–3. doi: 10.1016/j.it.2025.06.007 40683778

[66] Malviya V Yshii L Junius S Garg AD Humblet-Baron S Schlenner SM . Regulatory T-cell stability and functional plasticity in health and disease. Immunol Cell Biol. (2023) 101:112–29. doi: 10.1111/imcb.12613 36479949

[67] Hu W Dolsten GA Wang EY Beroshvili G Wang ZM Ghelani AP . Temporal and context-dependent requirements for the transcription factor Foxp3 expression in regulatory T cells. Nat Immunol. (2025) 26:2059–73. doi: 10.1038/s41590-025-02295-4 41062655 PMC12571910

[68] Cheng G Yu A Malek TR . T-cell tolerance and the multi-functional role of IL-2R signaling in T-regulatory cells. Immunol Rev. (2011) 241:63–76. doi: 10.1111/j.1600-065X.2011.01004.x 21488890 PMC3101713

[69] Shouse AN LaPorte KM Malek TR . Interleukin-2 signaling in the regulation of T cell biology in autoimmunity and cancer. Immunity. (2024) 57:414–28. doi: 10.1016/j.immuni.2024.02.001 38479359 PMC11126276

[70] Meguri Y Asano T Yoshioka T Iwamoto M Ikegawa S Sugiura H . Responses of regulatory and effector T-cells to low-dose interleukin-2 differ depending on the immune environment after allogeneic stem cell transplantation. Front Immunol. (2022) 13:891925. doi: 10.3389/fimmu.2022.891925 35983059 PMC9379320

[71] Chinen T Kannan AK Levine AG Fan X Klein U Zheng Y . An essential role for the IL-2 receptor in T(reg) cell function. Nat Immunol. (2016) 17:1322–33. doi: 10.1038/ni.3540 27595233 PMC5071159

[72] Solomou EE Juang YT Gourley MF Kammer GM Tsokos GC . Molecular basis of deficient IL-2 production in T cells from patients with systemic lupus erythematosus. J Immunol. (2001) 166:4216–22. doi: 10.4049/jimmunol.166.6.4216 11238674

[73] Hedrich CM Crispin JC Rauen T Ioannidis C Apostolidis SA Lo MS . cAMP response element modulator alpha controls IL2 and IL17A expression during CD4 lineage commitment and subset distribution in lupus. Proc Natl Acad Sci USA. (2012) 109:16606–11. doi: 10.1073/pnas.1210129109 23019580 PMC3478624

[74] Juang YT Wang Y Solomou EE Li Y Mawrin C Tenbrock K . Systemic lupus erythematosus serum IgG increases CREM binding to the IL-2 promoter and suppresses IL-2 production through CaMKIV. J Clin Invest. (2005) 115:996–1005. doi: 10.1172/JCI22854 15841182 PMC1070410

[75] Koga T Hedrich CM Mizui M Yoshida N Otomo K Lieberman LA . CaMK4-dependent activation of AKT/mTOR and CREM-alpha underlies autoimmunity-associated Th17 imbalance. J Clin Invest. (2014) 124:2234–45. doi: 10.1172/JCI73411 24667640 PMC4001553

[76] Hisada R Yoshida N Umeda M Burbano C Bhargava R Scherlinger M . The deacetylase SIRT2 contributes to autoimmune disease pathogenesis by modulating IL-17A and IL-2 transcription. Cell Mol Immunol. (2022) 19:738–50. doi: 10.1038/s41423-022-00874-1 35523941 PMC9151795

[77] Pan W Sharabi A Ferretti A Zhang Y Burbano C Yoshida N . PPP2R2D suppresses IL-2 production and Treg function. JCI Insight. (2020) 5:e138215. doi: 10.1172/jci.insight.138215 32897879 PMC7566706

[78] Katsiari CG Kyttaris VC Juang YT Tsokos GC . Protein phosphatase 2A is a negative regulator of IL-2 production in patients with systemic lupus erythematosus. J Clin Invest. (2005) 115:3193–204. doi: 10.1172/JCI24895 16224536 PMC1253625

[79] Sunahori K Juang YT Kyttaris VC Tsokos GC . Promoter hypomethylation results in increased expression of protein phosphatase 2A in T cells from patients with systemic lupus erythematosus. J Immunol. (2011) 186:4508–17. doi: 10.4049/jimmunol.1000340 21346232 PMC3148941

[80] Kato H Perl A . Mechanistic target of rapamycin complex 1 expands Th17 and IL-4+ CD4-CD8- double-negative T cells and contracts regulatory T cells in systemic lupus erythematosus. J Immunol. (2014) 192:4134–44. doi: 10.4049/jimmunol.1301859 24683191 PMC3995867

[81] Lai ZW Borsuk R Shadakshari A Yu J Dawood M Garcia R . Mechanistic target of rapamycin activation triggers IL-4 production and necrotic death of double-negative T cells in patients with systemic lupus erythematosus. J Immunol. (2013) 191:2236–46. doi: 10.4049/jimmunol.1301005 23913957 PMC3777662

[82] Yu Y Liu Y Shi FD Zou H Matarese G La Cava A . Cutting edge: Leptin-induced RORgammat expression in CD4+ T cells promotes Th17 responses in systemic lupus erythematosus. J Immunol. (2013) 190:3054–8. doi: 10.4049/jimmunol.1203275 23447682 PMC3608794

[83] Roth TL Puig-Saus C Yu R Shifrut E Carnevale J Li PJ . Reprogramming human T cell function and specificity with non-viral genome targeting. Nature. (2018) 559:405–9. doi: 10.1038/s41586-018-0326-5 29995861 PMC6239417

[84] Zhang Z Gothe F Pennamen P James JR McDonald D Mata CP . Human interleukin-2 receptor beta mutations associated with defects in immunity and peripheral tolerance. J Exp Med. (2019) 216:1311–27. doi: 10.1084/jem.20182304 31040185 PMC6547869

[85] Sharfe N Dadi HK Shahar M Roifman CM . Human immune disorder arising from mutation of the alpha chain of the interleukin-2 receptor. Proc Natl Acad Sci USA. (1997) 94:3168–71. doi: 10.1073/pnas.94.7.3168 9096364 PMC20340

[86] Apostolidis SA Rodriguez-Rodriguez N Suarez-Fueyo A Dioufa N Ozcan E Crispin JC . Phosphatase PP2A is requisite for the function of regulatory T cells. Nat Immunol. (2016) 17:556–64. doi: 10.1038/ni.3390 26974206 PMC4837024

[87] Vichos T Li W Karino K Rubin L Tsokos MG Pan W . ADAM9 supports regulatory T cell stability through TGF-β-SMAD signaling and is reduced in systemic lupus erythematosus. Clin Immunol. (2026) 285:110687. doi: 10.1016/j.clim.2026.110687 PMC13107534 41765214

[88] Ding Y Yu A Tsokos GC Malek TR . CD25 and protein phosphatase 2A cooperate to enhance IL-2R signaling in human regulatory T cells. J Immunol. (2019) 203:93–104. doi: 10.4049/jimmunol.1801570 31085588 PMC6581624

[89] Zemmour D Charbonnier LM Leon J Six E Keles S Delville M . Single-cell analysis of FOXP3 deficiencies in humans and mice unmasks intrinsic and extrinsic CD4(+) T cell perturbations. Nat Immunol. (2021) 22:607–19. doi: 10.1038/s41590-021-00910-8 33833438 PMC8173714

[90] Remedios KA Zirak B Sandoval PM Lowe MM Boda D Henley E . The TNFRSF members CD27 and OX40 coordinately limit T(H)17 differentiation in regulatory T cells. Sci Immunol. (2018) 3:eaau2042. doi: 10.1126/sciimmunol.aau2042 30578350

[91] Li L Kim J Boussiotis VA . IL-1beta-mediated signals preferentially drive conversion of regulatory T cells but not conventional T cells into IL-17-producing cells. J Immunol. (2010) 185:4148–53. doi: 10.4049/jimmunol.1001536 20817874 PMC2956066

[92] Hwang SM Sharma G Verma R Byun S Rudra D Im SH . Inflammation-induced Id2 promotes plasticity in regulatory T cells. Nat Commun. (2018) 9:4736. doi: 10.1038/s41467-018-07254-2 30413714 PMC6226514

[93] Zhou X Bailey-Bucktrout SL Jeker LT Penaranda C Martinez-Llordella M Ashby M . Instability of the transcription factor Foxp3 leads to the generation of pathogenic memory T cells *in vivo*. Nat Immunol. (2009) 10:1000–7. doi: 10.1038/ni.1774 19633673 PMC2729804

[94] Zong Y Deng K Chong WP . Regulation of Treg cells by cytokine signaling and co-stimulatory molecules. Front Immunol. (2024) 15:1387975. doi: 10.3389/fimmu.2024.1387975 38807592 PMC11131382

[95] Schnell JT Briviesca RL Kim T Charbonnier LM Henderson LA van Wijk F . The 'T(reg) paradox' in inflammatory arthritis. Nat Rev Rheumatol. (2025) 21:9–21. doi: 10.1038/s41584-024-01190-w 39653758

[96] Komatsu N Okamoto K Sawa S Nakashima T Oh-hora M Kodama T . Pathogenic conversion of Foxp3+ T cells into TH17 cells in autoimmune arthritis. Nat Med. (2014) 20:62–8. doi: 10.1038/nm.3432 24362934

[97] Nie H Zheng Y Li R Guo TB He D Fang L . Phosphorylation of FOXP3 controls regulatory T cell function and is inhibited by TNF-alpha in rheumatoid arthritis. Nat Med. (2013) 19:322–8. doi: 10.1038/nm.3085 23396208

[98] Hao Y Cook MC . Inborn errors of immunity and their phenocopies: CTLA4 and PD-1. Front Immunol. (2021) 12:806043. doi: 10.3389/fimmu.2021.806043 35154081 PMC8832511

[99] Tangye SG Al-Herz W Bousfiha A Cunningham-Rundles C Franco JL Holland SM . Human inborn errors of immunity: 2022 update on the classification from the International Union of Immunological Societies Expert Committee. J ClinImmunol. (2022) 42:1473–507. doi: 10.1007/s10875-022-01289-3 35748970 PMC9244088

[100] Zammit NW Siggs OM Gray PE Horikawa K Langley DB Walters SN . Denisovan, modern human and mouse TNFAIP3 alleles tune A20 phosphorylation and immunity. Nat Immunol. (2019) 20:1299–310. doi: 10.1038/s41590-019-0492-0 31534238

[101] Zammit NW Gray PE Siggs OM Yap JY Russell A Cultrone D . Environmental and genetic disease modifiers of haploinsufficiency of A20. bioRxiv. (2022), 2022.2003.2019.485004. doi: 10.1101/2022.03.19.48500438621210

[102] Zhou Q Wang H Schwartz DM Stoffels M Park YH Zhang Y . Loss-of-function mutations in TNFAIP3 leading to A20 haploinsufficiency cause an early-onset autoinflammatory disease. Nat Genet. (2016) 48:67–73. doi: 10.1038/ng.3459 26642243 PMC4777523

[103] Yang S Svensson MND Harder NHO Hsieh WC Santelli E Kiosses WB . PTPN22 phosphorylation acts as a molecular rheostat for the inhibition of TCR signaling. Sci Signal. (2020) 13:eaaw8130. doi: 10.1126/scisignal.aaw8130 32184287 PMC7263369

[104] Kolios AGA Tsokos GC Klatzmann D . Interleukin-2 and regulatory T cells in rheumatic diseases. Nat Rev Rheumatol. (2021) 17:749–66. doi: 10.1038/s41584-021-00707-x 34728817

[105] Chen YY Jeffery HC Hunter S Bhogal R Birtwistle J Braitch MK . Human intrahepatic regulatory T cells are functional, require IL-2 from effector cells for survival, and are susceptible to Fas ligand-mediated apoptosis. Hepatology. (2016) 64:138–50. doi: 10.1002/hep.28517 26928938 PMC4950043

[106] Arterbery AS Osafo-Addo A Avitzur Y Ciarleglio M Deng Y Lobritto SJ . Production of proinflammatory cytokines by monocytes in liver-transplanted recipients with de novo autoimmune hepatitis is enhanced and induces TH1-like regulatory T cells. J Immunol. (2016) 196:4040–51. doi: 10.4049/jimmunol.1502276 27183637 PMC4874532

[107] Goldberg R Clough JN Roberts LB Sanchez J Kordasti S Petrov N . A Crohn's disease-associated IL2RA enhancer variant determines the balance of T cell immunity by regulating responsiveness to IL-2 signalling. J Crohns Colitis. (2021) 15:2054–65. doi: 10.1093/ecco-jcc/jjab103 34120187 PMC8684452

[108] Cao AT Yao S Stefka AT Liu Z Qin H Liu H . TLR4 regulates IFN-gamma and IL-17 production by both thymic and induced Foxp3+ Tregs during intestinal inflammation. J Leukoc Biol. (2014) 96:895–905. doi: 10.1189/jlb.3A0114-056RR 25015957 PMC4197555

[109] Dominguez-Villar M Baecher-Allan CM Hafler DA . Identification of T helper type 1-like, Foxp3+ regulatory T cells in human autoimmune disease. Nat Med. (2011) 17:673–5. doi: 10.1038/nm.2389 21540856 PMC3675886

[110] Martin JE Carmona FD Broen JC Simeon CP Vonk MC Carreira P . The autoimmune disease-associated IL2RA locus is involved in the clinical manifestations of systemic sclerosis. Genes Immun. (2012) 13:191–6. doi: 10.1038/gene.2011.72 22012429

[111] Viglietta V Baecher-Allan C Weiner HL Hafler DA . Loss of functional suppression by CD4+CD25+ regulatory T cells in patients with multiple sclerosis. J Exp Med. (2004) 199:971–9. doi: 10.1084/jem.20031579 15067033 PMC2211881

[112] Bailey-Bucktrout SL Martinez-Llordella M Zhou X Anthony B Rosenthal W Luche H . Self-antigen-driven activation induces instability of regulatory T cells during an inflammatory autoimmune response. Immunity. (2013) 39:949–62. doi: 10.1016/j.immuni.2013.10.016 24238343 PMC3912996

[113] Moulton VR Tsokos GC . T cell signaling abnormalities contribute to aberrant immune cell function and autoimmunity. J Clin Invest. (2015) 125:2220–7. doi: 10.1172/JCI78087 25961450 PMC4497749

[114] Cerosaletti K Schneider A Schwedhelm K Frank I Tatum M Wei S . Multiple autoimmune-associated variants confer decreased IL-2R signaling in CD4+ CD25(hi) T cells of type 1 diabetic and multiple sclerosis patients. PloS One. (2013) 8:e83811. doi: 10.1371/journal.pone.0083811 24376757 PMC3871703

[115] Hulme MA Wasserfall CH Atkinson MA Brusko TM . Central role for interleukin-2 in type 1 diabetes. Diabetes. (2012) 61:14–22. doi: 10.2337/db11-1213 22187370 PMC3237657

[116] Hull CM Peakman M Tree TIM . Regulatory T cell dysfunction in type 1 diabetes: what's broken and how can we fix it? Diabetologia. (2017) 60:1839–50. doi: 10.1007/s00125-017-4377-1 28770318 PMC6448885

[117] Tang Q Adams JY Penaranda C Melli K Piaggio E Sgouroudis E . Central role of defective interleukin-2 production in the triggering of islet autoimmune destruction. Immunity. (2008) 28:687–97. doi: 10.1016/j.immuni.2008.03.016 18468463 PMC2394854

[118] McClymont SA Putnam AL Lee MR Esensten JH Liu W Hulme MA . Plasticity of human regulatory T cells in healthy subjects and patients with type 1 diabetes. J Immunol. (2011) 186:3918–26. doi: 10.4049/jimmunol.1003099 21368230 PMC3091943

[119] Sumida TS Cheru NT Hafler DA . The regulation and differentiation of regulatory T cells and their dysfunction in autoimmune diseases. Nat Rev Immunol. (2024) 24:503–17. doi: 10.1038/s41577-024-00994-x 38374298 PMC11216899

[120] Dendrou CA Plagnol V Fung E Yang JH Downes K Cooper JD . Cell-specific protein phenotypes for the autoimmune locus IL2RA using a genotype-selectable human bioresource. Nat Genet. (2009) 41:1011–5. doi: 10.1038/ng.434 19701192 PMC2749506

[121] Lowe CE Cooper JD Brusko T Walker NM Smyth DJ Bailey R . Large-scale genetic fine mapping and genotype-phenotype associations implicate polymorphism in the IL2RA region in type 1 diabetes. Nat Genet. (2007) 39:1074–82. doi: 10.1038/ng2102 17676041

[122] Todd JA Walker NM Cooper JD Smyth DJ Downes K Plagnol V . Robust associations of four new chromosome regions from genome-wide analyses of type 1 diabetes. Nat Genet. (2007) 39:857–64. doi: 10.1038/ng2068 17554260 PMC2492393

[123] Lawson JM Tremble J Dayan C Beyan H Leslie RD Peakman M . Increased resistance to CD4+CD25hi regulatory T cell-mediated suppression in patients with type 1 diabetes. Clin Exp Immunol. (2008) 154:353–9. doi: 10.1111/j.1365-2249.2008.03810.x 19037920 PMC2633239

[124] Schneider A Rieck M Sanda S Pihoker C Greenbaum C Buckner JH . The effector T cells of diabetic subjects are resistant to regulation via CD4+ FOXP3+ regulatory T cells. J Immunol. (2008) 181:7350–5. doi: 10.4049/jimmunol.181.10.7350 18981158 PMC2597079

[125] Dendrou CA Wicker LS . The IL-2/CD25 pathway determines susceptibility to T1D in humans and NOD mice. J ClinImmunol. (2008) 28:685–96. doi: 10.1007/s10875-008-9237-9 18780166

[126] Bayes B Pastor MC Lauzurica R Granada ML Salinas I Romero R . Do anti-CD25 monoclonal antibodies potentiate posttransplant diabetes mellitus? Transplant Proc. (2007) 39:2248–50. doi: 10.1016/j.transproceed.2007.06.021 17889153

[127] Vendrame F Pileggi A Laughlin E Allende G Martin-Pagola A Molano RD . Recurrence of type 1 diabetes after simultaneous pancreas-kidney transplantation, despite immunosuppression, is associated with autoantibodies and pathogenic autoreactive CD4 T-cells. Diabetes. (2010) 59:947–57. doi: 10.2337/db09-0498 20086230 PMC2844842

[128] Kumar M Yip L Wang F Marty SE Fathman CG . Autoimmune disease: genetic susceptibility, environmental triggers, and immune dysregulation. Where can we develop therapies? Front Immunol. (2025) 16:1626082. doi: 10.3389/fimmu.2025.1626082 40852720 PMC12367657

[129] Sheean RK McKay FC Cretney E Bye CR Perera ND Tomas D . Association of regulatory T-cell expansion with progression of amyotrophic lateral sclerosis: a study of humans and a transgenic mouse model. JAMA Neurol. (2018) 75:681–9. doi: 10.1001/jamaneurol.2018.0035 29507931 PMC5885208

[130] Saunders JA Estes KA Kosloski LM Allen HE Dempsey KM Torres-Russotto DR . CD4+ regulatory and effector/memory T cell subsets profile motor dysfunction in Parkinson's disease. J Neuroimmune Pharmacol. (2012) 7:927–38. doi: 10.1007/s11481-012-9402-z 23054369 PMC3515774

[131] Thome AD Atassi F Wang J Faridar A Zhao W Thonhoff JR . Ex vivo expansion of dysfunctional regulatory T lymphocytes restores suppressive function in Parkinson's disease. NPJ Parkinsons Dis. (2021) 7:41. doi: 10.1038/s41531-021-00188-5 33986285 PMC8119976

[132] Hu W Wang ZM Feng Y Schizas M Hoyos BE van der Veeken J . Regulatory T cells function in established systemic inflammation and reverse fatal autoimmunity. Nat Immunol. (2021) 22:1163–74. doi: 10.1038/s41590-021-01001-4 34426690 PMC9341271

[133] Allan SE Alstad AN Merindol N Crellin NK Amendola M Bacchetta R . Generation of potent and stable human CD4+ T regulatory cells by activation-independent expression of FOXP3. Mol Ther. (2008) 16:194–202. doi: 10.1038/sj.mt.6300341 17984976

[134] Henschel P Landwehr-Kenzel S Engels N Schienke A Kremer J Riet T . Supraphysiological FOXP3 expression in human CAR-Tregs results in improved stability, efficacy, and safety of CAR-Treg products for clinical application. J Autoimmun. (2023) 138:103057. doi: 10.1016/j.jaut.2023.103057 37224732

[135] Passerini L Rossi Mel E Sartirana C Fousteri G Bondanza A Naldini L . CD4(+) T cells from IPEX patients convert into functional and stable regulatory T cells by FOXP3 gene transfer. Sci Transl Med. (2013) 5:215ra174. doi: 10.1126/scitranslmed.3007320 24337481

[136] McGovern J Holler A Thomas S Stauss HJ . Forced Fox-P3 expression can improve the safety and antigen-specific function of engineered regulatory T cells. J Autoimmun. (2022) 132:102888. doi: 10.1016/j.jaut.2022.102888 36049437 PMC10570926

[137] Alexander J Agarwal R Alkayed A Ramachandran A Joseph K Ye J . Autologous lentiviral vector LVFOXP3 CD4 T cells for IPEX syndrome. J Hum Immun. (2025) 1:CIS2025abstract–2027. doi: 10.70962/cis2025abstract.27

[138] Honaker Y Hubbard N Xiang Y Fisher L Hagin D Sommer K . Gene editing to induce FOXP3 expression in human CD4(+) T cells leads to a stable regulatory phenotype and function. Sci Transl Med. (2020) 12:eaay6422. doi: 10.1126/scitranslmed.aay6422 32493794

[139] Hunt MS Yang SJ Mortensen E Boukhris A Buckner J Cook PJ . Dual-locus, dual-HDR editing permits efficient generation of antigen-specific regulatory T cells with robust suppressive activity. Mol Ther. (2023) 31:2872–86. doi: 10.1016/j.ymthe.2023.07.016 37481700 PMC10556186

[140] Uenishi GI Repic M Yam JY Landuyt A Saikumar-Lakshmi P Guo T . GNTI-122: an autologous antigen-specific engineered Treg cell therapy for type 1 diabetes. JCI Insight. (2024) 9:e171844. doi: 10.1172/jci.insight.171844 38516892 PMC11063937

[141] Hu W Dolsten G Beroshvili G Wang EY Wang ZM Ghelani AP . Temporal and Context-Dependent Requirements for the Transcription Factor Foxp3 Expression in Regulatory T Cells. Nat Immunol. (2025) 26(11):2059–73. doi: 10.1038/s41590-025-02295-4 PMC12571910 41062655

[142] van der Vliet HJ Nieuwenhuis EE . IPEX as a result of mutations in FOXP3. Clin Dev Immunol. (2007) 2007:89017. doi: 10.1155/2007/89017 18317533 PMC2248278

[143] Thornton AM Korty PE Tran DQ Wohlfert EA Murray PE Belkaid Y . Expression of Helios, an Ikaros transcription factor family member, differentiates thymic-derived from peripherally induced Foxp3+ T regulatory cells. J Immunol. (2010) 184:3433–42. doi: 10.4049/jimmunol.0904028 20181882 PMC3725574

[144] Seng A Krausz KL Pei D Koestler DC Fischer RT Yankee TM . Coexpression of FOXP3 and a Helios isoform enhances the effectiveness of human engineered regulatory T cells. Blood Adv. (2020) 4:1325–39. doi: 10.1182/bloodadvances.2019000965 32259202 PMC7160257

[145] Pan F Yu H Dang EV Barbi J Pan X Grosso JF . Eos mediates Foxp3-dependent gene silencing in CD4+ regulatory T cells. Science. (2009) 325:1142–6. doi: 10.1126/science.1176077 19696312 PMC2859703

[146] Xie X Thornton AM Tanwar S Korty P Wells AC Segrist E . Eos plays a critical role in Treg homeostasis and modulates the function of recirculating thymic Tregs in the control of Treg development. Cell Rep. (2026) 45:116838. doi: 10.1016/j.celrep.2025.116838 41474621 PMC12869356

[147] He M Xu B Bose PG McCullough MJ Sellers RS Zong X . T cell receptor signaling induces expression of lysine demethylase KDM6B to maintain Treg homeostasis. J Clin Invest. (2026) 136:e196022. doi: 10.1172/JCI196022 42024441 PMC13318106

[148] Fu W Ergun A Lu T Hill JA Haxhinasto S Fassett MS . A multiply redundant genetic switch 'locks in' the transcriptional signature of regulatory T cells. Nat Immunol. (2012) 13:972–80. doi: 10.1038/ni.2420 22961053 PMC3698954

[149] Yang F Yanes A Li M Heizer P Linatoc I Stephens ME . Differential regulation of Treg stability in human naive and effector Treg subsets by TGFbeta-signaling via ARKADIA-SKI axis. Front Immunol. (2025) 16:1636434. doi: 10.3389/fimmu.2025.1636434 40995370 PMC12454061

[150] Cortez JT Montauti E Shifrut E Gatchalian J Zhang Y Shaked O . CRISPR screen in regulatory T cells reveals modulators of Foxp3. Nature. (2020) 582:416–20. doi: 10.1038/s41586-020-2246-4 32499641 PMC7305989

[151] Chen KY Kibayashi T Giguelay A Hata M Nakajima S Mikami N . Genome-wide CRISPR screen in human T cells reveals regulators of FOXP3. Nature. (2025) 642:191–200. doi: 10.1038/s41586-025-08795-5 40140585

[152] Chen S Zhang L Ying Y Wang Y Arnold PR Wang G . Epigenetically modifying the Foxp3 locus for generation of stable antigen-specific Tregs as cellular therapeutics. Am J Transplant. (2020) 20:2366–79. doi: 10.1111/ajt.15845 32167228 PMC7483360

[153] Mikami N Kawakami R Sugimoto A Arai M Sakaguchi S . Generating functionally stable and antigen-specific T(reg) cells from effector T cells for cell therapy of inflammatory diseases. Sci Transl Med. (2025) 17:eadr6049. doi: 10.1126/scitranslmed.adr6049 41124283

[154] Selvaraj RK Geiger TL . A kinetic and dynamic analysis of Foxp3 induced in T cells by TGF-beta. J Immunol. (2007) 178:7667–77. doi: 10.4049/jimmunol.178.12.7667 17548603

[155] Wei J Duramad O Perng OA Reiner SL Liu YJ Qin FX . Antagonistic nature of T helper 1/2 developmental programs in opposing peripheral induction of Foxp3+ regulatory T cells. Proc Natl Acad Sci USA. (2007) 104:18169–74. doi: 10.1073/pnas.0703642104 17978190 PMC2084315

[156] Polansky JK Kretschmer K Freyer J Floess S Garbe A Baron U . DNA methylation controls Foxp3 gene expression. Eur J Immunol. (2008) 38:1654–63. doi: 10.1002/eji.200838105 18493985

[157] Akamatsu M Mikami N Ohkura N Kawakami R Kitagawa Y Sugimoto A . Conversion of antigen-specific effector/memory T cells into Foxp3-expressing T(reg) cells by inhibition of CDK8/19. Sci Immunol. (2019) 4:eaaw2707. doi: 10.1126/sciimmunol.aaw2707 31653719

[158] Kasahara H Kondo T Nakatsukasa H Chikuma S Ito M Ando M . Generation of allo-antigen-specific induced Treg stabilized by vitamin C treatment and its application for prevention of acute graft versus host disease model. Int Immunol. (2017) 29:457–69. doi: 10.1093/intimm/dxx060 29126272

[159] Nikolouli E Hardtke-Wolenski M Hapke M Beckstette M Geffers R Floess S . Alloantigen-induced regulatory T cells generated in presence of vitamin C display enhanced stability of foxp3 expression and promote skin allograft acceptance. Front Immunol. (2017) 8:748. doi: 10.3389/fimmu.2017.00748 28702031 PMC5487376

[160] Iamsawat S Tian L Daenthanasanmak A Wu Y Nguyen HD Bastian D . Vitamin C stabilizes CD8+ iTregs and enhances their therapeutic potential in controlling murine GVHD and leukemia relapse. Blood Adv. (2019) 3:4187–201. doi: 10.1182/bloodadvances.2019000531 31856270 PMC6929397

[161] Fontenot JD Rasmussen JP Gavin MA Rudensky AY . A function for interleukin 2 in Foxp3-expressing regulatory T cells. Nat Immunol. (2005) 6:1142–51. doi: 10.1038/ni1263 16227984

[162] Setoguchi R Hori S Takahashi T Sakaguchi S . Homeostatic maintenance of natural Foxp3(+) CD25(+) CD4(+) regulatory T cells by interleukin (IL)-2 and induction of autoimmune disease by IL-2 neutralization. J Exp Med. (2005) 201:723–35. doi: 10.1084/jem.20041982 15753206 PMC2212841

[163] Harris F Berdugo YA Tree T . IL-2-based approaches to Treg enhancement. Clin Exp Immunol. (2023) 211:149–63. doi: 10.1093/cei/uxac105 36399073 PMC10019135

[164] Amini L Kaeda J Weber O Reinke P . Low-dose interleukin-2 therapy: fine-tuning treg in solid organ transplantation? Transplantation. (2024) 108:1492–508. doi: 10.1097/TP.0000000000004866 38294829 PMC11188637

[165] Lykhopiy V Malviya V Humblet-Baron S Schlenner SM . IL-2 immunotherapy for targeting regulatory T cells in autoimmunity. Genes Immun. (2023) 24:248–62. doi: 10.1038/s41435-023-00221-y 37741949 PMC10575774

[166] Dixit N Fanton C Langowski JL Kirksey Y Kirk P Chang T . NKTR-358: A novel regulatory T-cell stimulator that selectively stimulates expansion and suppressive function of regulatory T cells for the treatment of autoimmune and inflammatory diseases. J Transl Autoimmun. (2021) 4:100103. doi: 10.1016/j.jtauto.2021.100103 34041473 PMC8141531

[167] Peterson LB Bell CJM Howlett SK Pekalski ML Brady K Hinton H . A long-lived IL-2 mutein that selectively activates and expands regulatory T cells as a therapy for autoimmune disease. J Autoimmun. (2018) 95:1–14. doi: 10.1016/j.jaut.2018.10.017 30446251 PMC6284106

[168] Fanton C Furie R Chindalore V Levin R Diab I Dixit N . Selective expansion of regulatory T cells by NKTR-358 in healthy volunteers and patients with systemic lupus erythematosus. J Transl Autoimmun. (2022) 5:100152. doi: 10.1016/j.jtauto.2022.100152 35517914 PMC9062472

[169] Silverberg JI Rosmarin D Chovatiya R Bieber T Schleicher S Beck L . The regulatory T cell-selective interleukin-2 receptor agonist rezpegaldesleukin in the treatment of inflammatory skin diseases: two randomized, double-blind, placebo-controlled phase 1b trials. Nat Commun. (2024) 15:9230. doi: 10.1038/s41467-024-53384-1 39455575 PMC11511931

[170] Yee C-S Gordon C Valenzuela G Karis E Wei J Jin YS . EFAVALEUKIN ALFA IN PATIENTS WITH ACTIVE SYSTEMIC LUPUS ERYTHEMATOSUS: RESULTS OF A BAYESIAN ADAPTIVE, PHASE 2B, DOUBLE-BLIND, RANDOMIZED, PLACEBO-CONTROLLED DOSE-RANGING STUDY. J Rheumatol. (2025) 52(253). doi: 10.3899/jrheum.2025-0390.PV277

[171] Sockolosky JT Trotta E Parisi G Picton L Su LL Le AC . Selective targeting of engineered T cells using orthogonal IL-2 cytokine-receptor complexes. Science. (2018) 359:1037–42. doi: 10.1126/science.aar3246 29496879 PMC5947856

[172] Indart A Lyu H Nguyen VQ Rosenthal W Jang SS Chen Y . Receptor-tethered orthogonal IL-2 enhances regulatory T cell therapy. bioRxiv. (2025) 223(9):e20251480. doi: 10.1101/2025.07.19.665688 42584417 PMC13521516

[173] Cook PJ Yang SJ Uenishi GI Grimm A West SE Wang LJ . A chemically inducible IL-2 receptor signaling complex allows for effective *in vitro* and *in vivo* selection of engineered CD4+ T cells. Mol Ther. (2023) 31:2472–88. doi: 10.1016/j.ymthe.2023.04.021 37147803 PMC10421999

[174] Choi J Chen J Schreiber SL Clardy J . Structure of the FKBP12-rapamycin complex interacting with the binding domain of human FRAP. Science. (1996) 273(5272):239–42. doi: 10.1126/science.273.5272.23 8662507

[175] Tewari R Yang SJ McClain ED Hu A Mortensen E DeSchmidt A . Identification of a novel PDC-E2 epitope in primary biliary cholangitis: Application for engineered Treg therapy. J Autoimmun. (2024) 149:103327. doi: 10.1016/j.jaut.2024.103327 39476446

[176] Bender C Wiedeman AE Hu A Ylescupidez A Sietsema WK Herold KC . A phase 2 randomized trial with autologous polyclonal expanded regulatory T cells in children with new-onset type 1 diabetes. Sci Transl Med. (2024) 16:eadn2404. doi: 10.1126/scitranslmed.adn2404 38718135

[177] Li MO Rudensky AY . T cell receptor signalling in the control of regulatory T cell differentiation and function. Nat Rev Immunol. (2016) 16:220–33. doi: 10.1038/nri.2016.26 27026074 PMC4968889

[178] Yeh WI Seay HR Newby B Posgai AL Moniz FB Michels A . Avidity and bystander suppressive capacity of human regulatory T cells expressing de novo autoreactive T-cell receptors in type 1 diabetes. Front Immunol. (2017) 8:1313. doi: 10.3389/fimmu.2017.01313 29123516 PMC5662552

[179] Yang SJ Singh AK Drow T Tappen T Honaker Y Barahmand-Pour-Whitman F . Pancreatic islet-specific engineered T(regs) exhibit robust antigen-specific and bystander immune suppression in type 1 diabetes models. Sci Transl Med. (2022) 14:eabn1716. doi: 10.1126/scitranslmed.abn1716 36197963

[180] Kim YC Zhang AH Yoon J Culp WE Lees JR Wucherpfennig KW . Engineered MBP-specific human Tregs ameliorate MOG-induced EAE through IL-2-triggered inhibition of effector T cells. J Autoimmun. (2018) 92:77–86. doi: 10.1016/j.jaut.2018.05.003 29857928 PMC6054915

[181] Kim YC Zhang A-H Su Y Rieder SA Rossi RJ Ettinger RA . Engineered antigen-specific human regulatory T cells: immunosuppression of FVIII-specific T-and B-cell responses. Blood J Am Soc Hematol. (2015) 125:1107–15. doi: 10.1182/blood-2014-04-566786 25498909 PMC4326771

[182] MacDonald KG Hoeppli RE Huang Q Gillies J Luciani DS Orban PC . Alloantigen-specific regulatory T cells generated with a chimeric antigen receptor. J Clin Invest. (2016) 126:1413–24. doi: 10.1172/JCI82771 26999600 PMC4811124

[183] Tripathi SK Grimm A Dahl NP Honaker Y Knebusch P Chen Y . HLA-A2 CAR/IL-2-CISC engineered Treg display robust *in vitro* and *in vivo* antigen-specific regulatory function. Mol Ther Methods Clin Dev. (2025) 33:101561. doi: 10.1016/j.omtm.2025.101561 40955314 PMC12433479

[184] Raffin C Muller Y Barragan J Zhou Y Piccoli L Lanzavecchia A . Development of citrullinated-vimentin-specific CAR for targeting Tregs to treat autoimmune rheumatoid arthritis. J Immunol. (2019) 202. doi: 10.4049/jimmunol.202.Supp.133.2

[185] De Paula Pohl A Schmidt A Zhang AH Maldonado T Konigs C Scott DW . Engineered regulatory T cells expressing myelin-specific chimeric antigen receptors suppress EAE progression. Cell Immunol. (2020) 358:104222. doi: 10.1016/j.cellimm.2020.104222 33053469

[186] Graber DJ Cook WJ Sentman ML Murad-Mabaera JM Sentman CL . Human CD4+CD25+ T cells expressing a chimeric antigen receptor against aberrant superoxide dismutase 1 trigger antigen-specific immunomodulation. Cytotherapy. (2024) 26:126–35. doi: 10.1016/j.jcyt.2023.11.007 38043051 PMC10872388

[187] Imura Y Ando M Kondo T Ito M Yoshimura A . CD19-targeted CAR regulatory T cells suppress B cell pathology without GvHD. JCI Insight. (2020) 5:e136185. doi: 10.1172/jci.insight.136185 32525846 PMC7453900

[188] Doglio M Ugolini A Bercher-Brayer C Camisa B Toma C Norata R . Regulatory T cells expressing CD19-targeted chimeric antigen receptor restore homeostasis in Systemic Lupus Erythematosus. Nat Commun. (2024) 15:2542. doi: 10.1038/s41467-024-46448-9 38538608 PMC10973480

[189] Salhotra A Wagner J Yang D Ali H Otoukesh S Juan J . First-in-human trial of allogeneic CD6-CAR tregs in patients with chronic graft-versus-host disease after allogeneic hematopoietic cell transplantation. Blood. (2025) 146:276. doi: 10.1182/blood-2025-276

[190] Pieper T Riet T Saetzler V Bergerhoff K McGovern J Delsing L . ENTPD3-specific CAR regulatory T cells for local immune control in T1D. bioRxiv. (2024). doi: 10.1101/2024.11.12.62295138621210

[191] Cui Y David M Bouchareychas L Rouquier S Sajuthi S Ayrault M . IL23R-specific CAR tregs for the treatment of crohn's disease. J Crohns Colitis. (2025) 19:jjae135. doi: 10.1093/ecco-jcc/jjae135 39252592 PMC11945296

[192] Rui X Alvarez Calderon F Wobma H Gerdemann U Albanese A Cagnin L . Human OX40L-CAR-T(regs) target activated antigen-presenting cells and control T cell alloreactivity. Sci Transl Med. (2024) 16:eadj9331. doi: 10.1126/scitranslmed.adj9331 39413160 PMC11789419

[193] Boardman DA Wong MQ Rees WD Wu D Himmel ME Orban PC . Flagellin-specific human CAR Tregs for immune regulation in IBD. J Autoimmun. (2023) 134:102961. doi: 10.1016/j.jaut.2022.102961 36470208 PMC9908852

[194] Arjomandnejad M Sylvia K Blackwood M Nixon T Tang Q Muhuri M . Modulating immune responses to AAV by expanded polyclonal T-regs and capsid specific chimeric antigen receptor T-regulatory cells. Mol Ther Methods Clin Dev. (2021) 23:490–506. doi: 10.1016/j.omtm.2021.10.010 34853797 PMC8605179

[195] Yoon J Schmidt A Zhang AH Konigs C Kim YC Scott DW . FVIII-specific human chimeric antigen receptor T-regulatory cells suppress T- and B-cell responses to FVIII. Blood. (2017) 129:238–45. doi: 10.1182/blood-2016-07-727834 28064157 PMC5234219

[196] Zhang AH Yoon J Kim YC Scott DW . Targeting antigen-specific B cells using antigen-expressing transduced regulatory T cells. J Immunol. (2018) 201:1434–41. doi: 10.4049/jimmunol.1701800 30021767 PMC6103823

[197] James EA Mallone R Kent SC DiLorenzo TP . T-cell epitopes and neo-epitopes in type 1 diabetes: A comprehensive update and reappraisal. Diabetes. (2020) 69:1311–35. doi: 10.2337/dbi19-0022 32561620 PMC7306130

[198] Michels AW Landry LG McDaniel KA Yu L Campbell-Thompson M Kwok WW . Islet-derived CD4 T cells targeting proinsulin in human autoimmune diabetes. Diabetes. (2017) 66:722–34. doi: 10.2337/db16-1025 27920090 PMC5319719

[199] Martinez-Llordella M Kenefeck R Seshadri S Mehtar F Voitovich A Burke C . Characterization of engineered CAR-Treg cells to induce immune tolerance in liver transplanted patients. Cytotherapy. (2025) 27:S41–2. doi: 10.1016/j.jcyt.2025.03.06842574925

[200] Schreeb K Atkinson G Chapman C Cao L Lu Y Meyer K . STEADFAST study update: A phase 1/2 clinical trial of regulatory T cells expressing a chimeric antigen receptor directed towards HLA-A2 in renal transplantation. Am J Transplant. (2025) 25:S265. doi: 10.1016/j.ajt.2025.07.58942574925

[201] Kohler M . A phase 1 study of autologous CAR-treg cells in refractory rheumatoid arthritis: interim report of safety and efficacy. ACR Convergence. (2025), 24–9.

[202] Lebrec H Maier CC Maki K Ponce R Shenton J Green S . Nonclinical safety assessment of engineered T cell therapies. Regul Toxicol Pharmacol. (2021) 127:105064. doi: 10.1016/j.yrtph.2021.105064 34656748

[203] Jadlowsky JK Hexner EO Marshall A Grupp SA Frey NV Riley JL . Long-term safety of lentiviral or gammaretroviral gene-modified T cell therapies. Nat Med. (2025) 31:1134–44. doi: 10.1038/s41591-024-03478-6 39833408

[204] Kalter N Fuster-Garcia C Silva A Ronco-Diaz V Roncelli S Turchiano G . Off-target effects in CRISPR-Cas genome editing for human therapeutics: Progress and challenges. Mol Ther Nucleic Acids. (2025) 36:102636. doi: 10.1016/j.omtn.2025.102636 40777742 PMC12329535

[205] Hsu C Jones SA Cohen CJ Zheng Z Kerstann K Zhou J . Cytokine-independent growth and clonal expansion of a primary human CD8+ T-cell clone following retroviral transduction with the IL-15 gene. Blood. (2007) 109:5168–77. doi: 10.1182/blood-2006-06-029173 17353346 PMC1890824

[206] Lamarche C Ward-Hartstonge K Mi T Lin DTS Huang Q Brown A . Tonic-signaling chimeric antigen receptors drive human regulatory T cell exhaustion. Proc Natl Acad Sci USA. (2023) 120:e2219086120. doi: 10.1073/pnas.2219086120 36972454 PMC10083618

[207] Long AH Haso WM Shern JF Wanhainen KM Murgai M Ingaramo M . 4-1BB costimulation ameliorates T cell exhaustion induced by tonic signaling of chimeric antigen receptors. Nat Med. (2015) 21:581–90. doi: 10.1038/nm.3838 25939063 PMC4458184

[208] Cochrane RW Robino RA Granger B Allen E Vaena S Romeo MJ . High-affinity chimeric antigen receptor signaling induces an inflammatory program in human regulatory T cells. Mol Ther Methods Clin Dev. (2024) 32:101385. doi: 10.1016/j.omtm.2024.101385 39687729 PMC11647616

[209] Honaker Y Dahl NP Singh S Singh AK Lopez C Gruber D . Preclinical efficacy and safety assessment of engineered regulatory T cells for treatment of IPEX and other autoimmune disorders. Mol Ther. (2026) 34:4035–53. doi: 10.1016/j.ymthe.2026.03.017 41832599 PMC13330026

[210] Jugder BE Park E Du L Jawale C Popov N Guo Z . Tissue-specific roles of regulatory T cells: mechanisms of suppression and beyond along with emerging therapeutic insights in autoimmune indications. Front Immunol. (2025) 16:1650451. doi: 10.3389/fimmu.2025.1650451 40933988 PMC12417136

[211] Feuerer M Gray DHD Rankin LC Schmidleithner L Liston A . Tissue regulatory T cells. Annu Rev Immunol. (2026) 44:237–65. doi: 10.1146/annurev-immunol-082924-092651 41678286

[212] Jayewickreme T Benoist C Mathis D . Lymph node stromal cell responses to perinatal T cell waves, a temporal atlas. Proc Natl Acad Sci USA. (2023) 120:e2316957120. doi: 10.1073/pnas.2316957120 38079541 PMC10740392

[213] Yaghi OK Hanna BS Langston PK Michelson DA Jayewickreme T Marin-Rodero M . A discrete 'early-responder' stromal-cell subtype orchestrates immunocyte recruitment to injured tissue. Nat Immunol. (2023) 24:2053–67. doi: 10.1038/s41590-023-01669-w 37932455 PMC10792729

[214] Haberman ER Sarker G Arus BA Ziegler KA Meunier S Martinez-Sanchez N . Immunomodulatory leptin receptor(+) sympathetic perineurial barrier cells protect against obesity by facilitating brown adipose tissue thermogenesis. Immunity. (2024) 57:141–52:e145. doi: 10.1016/j.immuni.2023.11.006 38091996

[215] Medrikova D Sijmonsma TP Sowodniok K Richards DM Delacher M Sticht C . Brown adipose tissue harbors a distinct sub-population of regulatory T cells. PloS One. (2015) 10:e0118534. doi: 10.1371/journal.pone.0118534 25714366 PMC4340926

[216] Kalin S Becker M Ott VB Serr I Hosp F Mollah MMH . A Stat6/Pten axis links regulatory T cells with adipose tissue function. Cell Metab. (2017) 26:475–92:e477. doi: 10.1016/j.cmet.2017.08.008 28877454 PMC5627977

[217] Langston PK Sun Y Ryback BA Mueller AL Spiegelman BM Benoist C . Regulatory T cells shield muscle mitochondria from interferon-gamma-mediated damage to promote the beneficial effects of exercise. Sci Immunol. (2023) 8:eadi5377. doi: 10.1126/sciimmunol.adi5377 37922340 PMC10860652

[218] Feuerer M Herrero L Cipolletta D Naaz A Wong J Nayer A . Lean, but not obese, fat is enriched for a unique population of regulatory T cells that affect metabolic parameters. Nat Med. (2009) 15:930–9. doi: 10.1038/nm.2002 19633656 PMC3115752

[219] Cipolletta D Cohen P Spiegelman BM Benoist C Mathis D . Appearance and disappearance of the mRNA signature characteristic of Treg cells in visceral adipose tissue: age, diet, and PPARgamma effects. Proc Natl Acad Sci USA. (2015) 112:482–7. doi: 10.1073/pnas.1423486112 25550516 PMC4299242

[220] Cipolletta D Feuerer M Li A Kamei N Lee J Shoelson SE . PPAR-gamma is a major driver of the accumulation and phenotype of adipose tissue Treg cells. Nature. (2012) 486:549–53. doi: 10.1038/nature11132 22722857 PMC3387339

[221] Vasanthakumar A Moro K Xin A Liao Y Gloury R Kawamoto S . The transcriptional regulators IRF4, BATF and IL-33 orchestrate development and maintenance of adipose tissue-resident regulatory T cells. Nat Immunol. (2015) 16:276–85. doi: 10.1038/ni.3085 25599561

[222] Spallanzani RG Zemmour D Xiao T Jayewickreme T Li C Bryce PJ . Distinct immunocyte-promoting and adipocyte-generating stromal components coordinate adipose tissue immune and metabolic tenors. Sci Immunol. (2019) 4:eaaw3658. doi: 10.1126/sciimmunol.aaw3658 31053654 PMC6648660

[223] Torres SV Man K Elmzzahi T Malko D Chisanga D Liao Y . Two regulatory T cell populations in the visceral adipose tissue shape systemic metabolism. Nat Immunol. (2024) 25:496–511. doi: 10.1038/s41590-024-01753-9 38356058

[224] Wu D Han JM Yu X Lam AJ Hoeppli RE Pesenacker AM . Characterization of regulatory T cells in obese omental adipose tissue in humans. Eur J Immunol. (2019) 49:336–47. doi: 10.1002/eji.201847570 30566246

[225] Wang G Munoz-Rojas AR Spallanzani RG Franklin RA Benoist C Mathis D . Adipose-tissue Treg cells restrain differentiation of stromal adipocyte precursors to promote insulin sensitivity and metabolic homeostasis. Immunity. (2024) 57:1345–59:e1345. doi: 10.1016/j.immuni.2024.04.002 38692280 PMC11188921

[226] A M Wang G Zammit NW Roth L Maierhaba Y Bogoslavski D . Irisin ameliorates obesity and insulin resistance via adipose tissue IL-33 and regulatory T cells. Nat Metab. (2026) 8:885–901. doi: 10.1038/s42255-026-01491-2 41933175 PMC13052164

[227] Hinte LC Castellano-Castillo D Ghosh A Melrose K Gasser E Noe F . Author correction: Adipose tissue retains an epigenetic memory of obesity after weight loss. Nature. (2025) 643:E24. doi: 10.1038/s41586-025-08909-z 40624358 PMC12286848

[228] Niven J Kucuk S Gope A Certo M Cassidy FC Arana Echarri A . DNA methylation-mediated memory of obesity in CD4 T lymphocytes perpetuates immune dysregulation. EMBO Rep. (2026) 27(11):3120–52. doi: 10.1038/s44319-026-00765-w 42045443 PMC13260840

[229] Munoz-Rojas AR Wang G Benoist C Mathis D . Adipose-tissue regulatory T cells are a consortium of subtypes that evolves with age and diet. Proc Natl Acad Sci USA. (2024) 121:e2320602121. doi: 10.1073/pnas.2320602121 38227656 PMC10823167

[230] Vasanthakumar A Chisanga D Blume J Gloury R Britt K Henstridge DC . Sex-specific adipose tissue imprinting of regulatory T cells. Nature. (2020) 579:581–5. doi: 10.1038/s41586-020-2040-3 32103173 PMC7241647

[231] Li Y Lu Y Lin SH Li N Han Y Huang Q . Insulin signaling establishes a developmental trajectory of adipose regulatory T cells. Nat Immunol. (2021) 22:1175–85. doi: 10.1038/s41590-021-01010-3 34429546

[232] Gnad T Navarro G Lahesmaa M Reverte-Salisa L Copperi F Cordomi A . Adenosine/A2B receptor signaling ameliorates the effects of aging and counteracts obesity. Cell Metab. (2020) 32:56–70:e57. doi: 10.1016/j.cmet.2020.06.006 32589947 PMC7437516

[233] Arpaia N Green JA Moltedo B Arvey A Hemmers S Yuan S . A distinct function of regulatory T cells in tissue protection. Cell. (2015) 162:1078–89. doi: 10.1016/j.cell.2015.08.021 26317471 PMC4603556

[234] Kaiser KA Loffredo LF Santos-Alexis KL Ringham OR Arpaia N . Regulation of the alveolar regenerative niche by amphiregulin-producing regulatory T cells. J Exp Med. (2023) 220:e20221462. doi: 10.1084/jem.20221462 36534084 PMC9767680

[235] Wang Y Li J Zhang Y He P Liu W Zeng W . AREG(+) regulatory T cells mediating myocardial repair and neovascularization after myocardial infarction. Mol Med. (2025) 31:229. doi: 10.1186/s10020-025-01281-8 40495143 PMC12153170

[236] Nosbaum A Prevel N Truong HA Mehta P Ettinger M Scharschmidt TC . Cutting edge: Regulatory T cells facilitate cutaneous wound healing. J Immunol. (2016) 196:2010–4. doi: 10.4049/jimmunol.1502139 26826250 PMC4761457

[237] Ito M Komai K Mise-Omata S Iizuka-Koga M Noguchi Y Kondo T . Brain regulatory T cells suppress astrogliosis and potentiate neurological recovery. Nature. (2019) 565:246–50. doi: 10.1038/s41586-018-0824-5 30602786

[238] Yao H Liu Y Wang Y Xue Y Jiang S Sun X . Dural Tregs driven by astrocytic IL-33 mitigate depression through the EGFR signals in mPFC neurons. Cell Death Differ. (2025) 32:926–43. doi: 10.1038/s41418-024-01421-3 39592709 PMC12089603

[239] Zaiss DM van Loosdregt J Gorlani A Bekker CP Grone A Sibilia M . Amphiregulin enhances regulatory T cell-suppressive function via the epidermal growth factor receptor. Immunity. (2013) 38:275–84. doi: 10.1016/j.immuni.2012.09.023 23333074 PMC3582723

[240] Liu Q Dwyer GK Zhao Y Li H Mathews LR Chakka AB . IL-33-mediated IL-13 secretion by ST2+ Tregs controls inflammation after lung injury. JCI Insight. (2019) 4:e123919. doi: 10.1172/jci.insight.123919 30779711 PMC6482994

[241] Harb H Benamar M Lai PS Contini P Griffith JW Crestani E . Notch4 signaling limits regulatory T-cell-mediated tissue repair and promotes severe lung inflammation in viral infections. Immunity. (2021) 54:1186–99:e1187. doi: 10.1016/j.immuni.2021.04.002 33915108 PMC8080416

[242] Deng B Wehling-Henricks M Villalta SA Wang Y Tidball JG . IL-10 triggers changes in macrophage phenotype that promote muscle growth and regeneration. J Immunol. (2012) 189:3669–80. doi: 10.4049/jimmunol.1103180 22933625 PMC3448810

[243] Panduro M Benoist C Mathis D . T(reg) cells limit IFN-gamma production to control macrophage accrual and phenotype during skeletal muscle regeneration. Proc Natl Acad Sci USA. (2018) 115:E2585–93. doi: 10.1073/pnas.1800618115 29476012 PMC5856564

[244] Villalta SA Rosenthal W Martinez L Kaur A Sparwasser T Tidball JG . Regulatory T cells suppress muscle inflammation and injury in muscular dystrophy. Sci Transl Med. (2014) 6:258ra142. doi: 10.1126/scitranslmed.3009925 25320234 PMC4889432

[245] Tang TT Yuan J Zhu ZF Zhang WC Xiao H Xia N . Regulatory T cells ameliorate cardiac remodeling after myocardial infarction. Basic Res Cardiol. (2012) 107:232. doi: 10.1007/s00395-011-0232-6 22189560

[246] Sharir R Semo J Shimoni S Ben-Mordechai T Landa-Rouben N Maysel-Auslender S . Experimental myocardial infarction induces altered regulatory T cell hemostasis, and adoptive transfer attenuates subsequent remodeling. PloS One. (2014) 9:e113653. doi: 10.1371/journal.pone.0113653 25436994 PMC4249913

[247] Xia N Lu Y Gu M Li N Liu M Jiao J . A unique population of regulatory T cells in heart potentiates cardiac protection from myocardial infarction. Circulation. (2020) 142:1956–73. doi: 10.1161/CIRCULATIONAHA.120.046789 32985264

[248] Weirather J Hofmann UD Beyersdorf N Ramos GC Vogel B Frey A . Foxp3+ CD4+ T cells improve healing after myocardial infarction by modulating monocyte/macrophage differentiation. Circ Res. (2014) 115:55–67. doi: 10.1161/CIRCRESAHA.115.303895 24786398

[249] Shi L Sun Z Su W Xu F Xie D Zhang Q . Treg cell-derived osteopontin promotes microglia-mediated white matter repair after ischemic stroke. Immunity. (2021) 54:1527–42:e1528. doi: 10.1016/j.immuni.2021.04.022 34015256 PMC8282725

[250] Hui SP Sheng DZ Sugimoto K Gonzalez-Rajal A Nakagawa S Hesselson D . Zebrafish regulatory T cells mediate organ-specific regenerative programs. Dev Cell. (2017) 43:659–72:e655. doi: 10.1016/j.devcel.2017.11.010 29257949

[251] Gupta S Adhikary S Hui SP . Decoding the proregenerative competence of regulatory T cells through complex tissue regeneration in zebrafish. Clin Exp Immunol. (2021) 206:346–53. doi: 10.1111/cei.13661 34529822 PMC8561694

[252] Cho I Lui PP Ali N . Treg regulation of the epithelial stem cell lineage. J Immunol Regener Med. (2020) 8:100028. doi: 10.1016/j.regen.2020.100028 32494759 PMC7226844

[253] Liu Z Hu X Liang Y Yu J Li H Shokhirev MN . Glucocorticoid signaling and regulatory T cells cooperate to maintain the hair-follicle stem-cell niche. Nat Immunol. (2022) 23:1086–97. doi: 10.1038/s41590-022-01244-9 35739197 PMC9283297

[254] Massague J Blain SW Lo RS . TGFbeta signaling in growth control, cancer, and heritable disorders. Cell. (2000) 103:295–309. doi: 10.1016/s0092-8674(00)00121-5 11057902

[255] Cohen JN Gouirand V Macon CE Lowe MM Boothby IC Moreau JM . Regulatory T cells in skin mediate immune privilege of the hair follicle stem cell niche. Sci Immunol. (2024) 9:eadh0152. doi: 10.1126/sciimmunol.adh0152 38181095 PMC11003870

[256] Zamfirescu AM Yatsenko AS Shcherbata HR . Notch signaling sculpts the stem cell niche. Front Cell Dev Biol. (2022) 10:1027222. doi: 10.3389/fcell.2022.1027222 36605720 PMC9810114

[257] Gonzalez R Reinberg D . The Notch pathway: A guardian of cell fate during neurogenesis. Curr Opin Cell Biol. (2025) 95:102543. doi: 10.1016/j.ceb.2025.102543 40450792 PMC12278768

[258] Veniaminova NA Grachtchouk M Doane OJ Peterson JK Quigley DA Lull MV . Niche-specific factors dynamically regulate sebaceous gland stem cells in the skin. Dev Cell. (2019) 51:326–40:e324. doi: 10.1016/j.devcel.2019.08.015 31564613 PMC6832854

[259] Blokzijl A Dahlqvist C Reissmann E Falk A Moliner A Lendahl U . Cross-talk between the Notch and TGF-beta signaling pathways mediated by interaction of the Notch intracellular domain with Smad3. J Cell Biol. (2003) 163:723–8. doi: 10.1083/jcb.200305112 14638857 PMC2173673

[260] Zavadil J Cermak L Soto-Nieves N Bottinger EP . Integration of TGF-beta/Smad and Jagged1/Notch signalling in epithelial-to-mesenchymal transition. EMBO J. (2004) 23:1155–65. doi: 10.1038/sj.emboj.7600069 14976548 PMC380966

[261] Fu Y Chang A Chang L Niessen K Eapen S Setiadi A . Differential regulation of transforming growth factor beta signaling pathways by Notch in human endothelial cells. J Biol Chem. (2009) 284:19452–62. doi: 10.1074/jbc.M109.011833 19473993 PMC2740571

[262] Tang Y Urs S Boucher J Bernaiche T Venkatesh D Spicer DB . Notch and transforming growth factor-beta (TGFbeta) signaling pathways cooperatively regulate vascular smooth muscle cell differentiation. J Biol Chem. (2010) 285:17556–63. doi: 10.1074/jbc.M109.076414 20368328 PMC2878520

[263] Camacho V Matkins VR Patel SB Lever JM Yang Z Ying L . Bone marrow Tregs mediate stromal cell function and support hematopoiesis via IL-10. JCI Insight. (2020) 5:e135681. doi: 10.1172/jci.insight.135681 33208555 PMC7710301

[264] Chen R Zhang X Li B Tonetti MS Yang Y Li Y . Progranulin-dependent repair function of regulatory T cells drives bone-fracture healing. J Clin Invest. (2024) 135:e164091. doi: 10.1172/JCI180679 39509336 PMC11735098

[265] Marin-Rodero M Cintado E Walker AJ Jayewickreme T Pinho-Ribeiro FA Richardson Q . The meninges host a distinct compartment of regulatory T cells that preserves brain homeostasis. Sci Immunol. (2025) 10:eadu2910. doi: 10.1126/sciimmunol.adu2910 39873623 PMC11924117

[266] Wang J Xie L Yang C Ren C Zhou K Wang B . Activated regulatory T cell regulates neural stem cell proliferation in the subventricular zone of normal and ischemic mouse brain through interleukin 10. Front Cell Neurosci. (2015) 9:361. doi: 10.3389/fncel.2015.00361 26441532 PMC4568339

[267] Dombrowski Y O'Hagan T Dittmer M Penalva R Mayoral SR Bankhead P . Regulatory T cells promote myelin regeneration in the central nervous system. Nat Neurosci. (2017) 20:674–80. doi: 10.1038/nn.4528 28288125 PMC5409501

[268] Delacher M Simon M Sanderink L Hotz-Wagenblatt A Wuttke M Schambeck K . Single-cell chromatin accessibility landscape identifies tissue repair program in human regulatory T cells. Immunity. (2021) 54:702–20:e717. doi: 10.1016/j.immuni.2021.03.007 33789089 PMC8050210

[269] Li C Munoz-Rojas AR Wang G Mann AO Benoist C Mathis D . PPARgamma marks splenic precursors of multiple nonlymphoid-tissue Treg compartments. Proc Natl Acad Sci USA. (2021) 118:e2025197118. doi: 10.1073/pnas.2025197118 33753509 PMC8020779

[270] Burton OT Bricard O Tareen S Gergelits V Andrews S Biggins L . The tissue-resident regulatory T cell pool is shaped by transient multi-tissue migration and a conserved residency program. Immunity. (2024) 57:1586–602:e1510. doi: 10.1016/j.immuni.2024.05.023 38897202

[271] Dwyer GK D'Cruz LM Turnquist HR . Emerging functions of IL-33 in homeostasis and immunity. Annu Rev Immunol. (2022) 40:15–43. doi: 10.1146/annurev-immunol-101320-124243 34985928

[272] Stremska ME Jose S Sabapathy V Huang L Bajwa A Kinsey GR . IL233, a novel IL-2 and IL-33 hybrid cytokine, ameliorates renal injury. J Am Soc Nephrol. (2017) 28:2681–93. doi: 10.1681/ASN.2016121272 28539382 PMC5576940

[273] Kawai K Uchiyama M Hester J Issa F . IL-33 drives the production of mouse regulatory T cells with enhanced *in vivo* suppressive activity in skin transplantation. Am J Transplant. (2021) 21:978–92. doi: 10.1111/ajt.16266 33314772 PMC7613121

[274] Ganchiku Y Ribas GT Lima K Gassen RB Liu K Efe O . IL-2 mutein promotes antigen-specific transplant acceptance in mice through expansion of ST2(+) regulatory T cells. Nat Commun. (2026) 17. doi: 10.1038/s41467-026-74185-8 42331795 PMC13287734

[275] Poder J Yu A Ding Y Albalaa C Klatzmann D Villarino AV . Durable control of autoimmunity requires sustained stimulation of regulatory T cells. Cell Rep. (2026) 45:117292. doi: 10.1016/j.celrep.2026.117292 42012980

[276] Pastille E Wasmer MH Adamczyk A Vu VP Mager LF Phuong NNT . The IL-33/ST2 pathway shapes the regulatory T cell phenotype to promote intestinal cancer. Mucosal Immunol. (2019) 12:990–1003. doi: 10.1038/s41385-019-0176-y 31165767 PMC7746527

[277] Lam AJ MacDonald KN Pesenacker AM Juvet SC Morishita KA Bressler B . Innate control of tissue-reparative human regulatory T cells. J Immunol. (2019) 202:2195–209. doi: 10.4049/jimmunol.1801330 30850479

[278] Mensink M Verleng LJ Schrama E Janssen GM Tjokrodirijo RT van Veelen PA . Tregs from human blood differentiate into nonlymphoid tissue-resident effector cells upon TNFR2 costimulation. JCI Insight. (2024) 9:e172942. doi: 10.1172/jci.insight.172942 38341270 PMC10972588

[279] Fierer J Kirkland T Finley F . Comparison of fluconazole and SDZ89-485 for therapy of experimental murine coccidioidomycosis. Antimicrob Agents Chemother. (1990) 34:13–6. doi: 10.1128/AAC.34.1.13 2327748 PMC171511

[280] de Kivit S Mensink M Hoekstra AT Berlin I Derks RJE Both D . Stable human regulatory T cells switch to glycolysis following TNF receptor 2 costimulation. Nat Metab. (2020) 2:1046–61. doi: 10.1038/s42255-020-00271-w 32958937

[281] Mensink M Tran TNM Zaal EA Schrama E Berkers CR Borst J . TNFR2 costimulation differentially impacts regulatory and conventional CD4(+) T-cell metabolism. Front Immunol. (2022) 13:881166. doi: 10.3389/fimmu.2022.881166 35844585 PMC9282886

[282] Chen X Baumel M Mannel DN Howard OM Oppenheim JJ . Interaction of TNF with TNF receptor type 2 promotes expansion and function of mouse CD4+CD25+ T regulatory cells. J Immunol. (2007) 179:154–61. doi: 10.4049/jimmunol.179.1.154 17579033

[283] Chopra M Biehl M Steinfatt T Brandl A Kums J Amich J . Exogenous TNFR2 activation protects from acute GvHD via host T reg cell expansion. J Exp Med. (2016) 213:1881–900. doi: 10.1084/jem.20151563 27526711 PMC4995078

[284] Leclerc M Naserian S Pilon C Thiolat A Martin GH Pouchy C . Control of GVHD by regulatory T cells depends on TNF produced by T cells and TNFR2 expressed by regulatory T cells. Blood. (2016) 128:1651–9. doi: 10.1182/blood-2016-02-700849 27506541

[285] Chen X Wu X Zhou Q Howard OM Netea MG Oppenheim JJ . TNFR2 is critical for the stabilization of the CD4+Foxp3+ regulatory T. cell phenotype in the inflammatory environment. J Immunol. (2013) 190:1076–84. doi: 10.4049/jimmunol.1202659 23277487 PMC3552130

[286] DiSpirito JR Zemmour D Ramanan D Cho J Zilionis R Klein AM . Molecular diversification of regulatory T cells in nonlymphoid tissues. Sci Immunol. (2018) 3:eaat5861. doi: 10.1126/sciimmunol.aat5861 30217811 PMC6219455

[287] Chowdhary K Leon J Mathis D Benoist C . An integrated transcription factor framework for Treg identity and diversity. Proc Natl Acad Sci USA. (2024) 121:e2411301121. doi: 10.1073/pnas.2411301121 39196621 PMC11388289

